# Delta/Notch signaling in glia maintains motor nerve barrier function and synaptic transmission by controlling matrix metalloproteinase expression

**DOI:** 10.1073/pnas.2110097119

**Published:** 2022-08-15

**Authors:** Mario R. Calderon, Megumi Mori, Grant Kauwe, Jill Farnsworth, Suzana Ulian-Benitez, Elie Maksoud, Jordan Shore, A. Pejmun Haghighi

**Affiliations:** ^a^Buck Institute for Research on Aging, Novato, CA 94945;; ^b^Leonard Davis School of Gerontology, University of Southern California, Los Angeles, CA 90089

**Keywords:** blood–brain barrier, glia, synaptic function, Delta/Notch signaling, metalloproteinase

## Abstract

While the role of barrier function in establishing a protective, nutrient-rich, and ionically balanced environment for neurons has been appreciated for some time, little is known about how signaling cues originating in barrier-forming cells participate in maintaining barrier function and influence synaptic activity. We have identified Delta/Notch signaling in subperineurial glia (SPG), a crucial glial type for *Drosophila* motor axon ensheathment and the blood–brain barrier, to be essential for controlling the expression of matrix metalloproteinase 1 (Mmp1), a major regulator of the extracellular matrix (ECM). Our genetic analysis indicates that Delta/Notch signaling in SPG exerts an inhibitory control on Mmp1 expression. In the absence of this inhibition, abnormally enhanced Mmp1 activity disrupts septate junctions and glial ensheathment of peripheral motor nerves, compromising neurotransmitter release at the neuromuscular junction (NMJ). Temporally controlled and cell type–specific transgenic analysis shows that Delta/Notch signaling inhibits transcription of Mmp1 by inhibiting c-Jun N-terminal kinase (JNK) signaling in SPG. Our results provide a mechanistic insight into the regulation of neuronal health and function via glial-initiated signaling and open a framework for understanding the complex relationship between ECM regulation and the maintenance of barrier function.

## Significance

We have made a surprising discovery linking Delta/Notch signaling in subperineurial glia (SPG) to the regulation of nerve ensheathment and neurotransmitter release at the *Drosophila* neuromuscular junction (NMJ). SPG, the counterpart of the endothelial layer in the vertebrate blood–brain barrier, form the key cellular layer that is critical for axonal ensheathment and the blood–brain barrier in *Drosophila*. Our findings demonstrate that Delta/Notch signaling exerts a constitutive negative inhibition on JNK signaling in SPG, thereby limiting the expression of Mmp1, a matrix metalloproteinase. SPG-specific and temporally regulated knockdown of Delta leads to breakdown of barrier function and compromises neurotransmitter release at the NMJ. Our results provide a mechanistic insight into the biology of barrier function and glia–neuron interactions.

Sophisticated neuronal functions require balanced ionic exchange, a steady supply of metabolites and nutrients, and protection against toxins and pathogens (1). Glia have emerged as key players in both the vertebrate and invertebrate nervous systems by providing many of these essential functions to neurons, highlighting the importance of glia–neuron interactions. As a result, cues that regulate how glia and neurons interact can potentially have profound consequences for the functioning of the nervous system.

In the *Drosophila* peripheral nervous system (PNS), motor axons are ensheathed by three layers of glia. The first ensheathing layer is formed by peripheral wrapping glia (WG) that closely parallels Remak bundle ensheathment in the mammalian system (2). The nerve is then enveloped by two additional concentric glial layers composed of subperineurial glia (SPG) and perineurial glia (PG), and finally a specialized dense extracellular matrix (ECM) called the neural lamella (NL) forms the outermost layer (3–5). Paracellular diffusion is blocked by a single autocellular junction formed by the septate junctions (SJs) of SPG cells (5–8). Malformed SJs or defective glial ensheathment by WG at peripheral nerves impairs axonal conduction and leads to uncoordinated muscle contractions (9–12). Glial ensheathment of *Drosophila* peripheral motor nerves requires the biosynthesis of ceramide-phosphoethanolamine, glial-initiated kinase signaling, and, like Schwann cell ensheathment of mammalian motor axons, ECM-initiated cues (11, 13–20). However, unlike the mammalian neuromuscular junction (NMJ), which is a tripartite synapse composed of motor nerve terminals, postjunctional muscle membranes, and terminal Schwann cells (21), the *Drosophila* NMJ is only partially and transiently engulfed by PG and SPG processes (7, 22, 23).

In both vertebrates and invertebrates, matrix metalloproteinases (Mmps), a class of zinc-dependent endopeptidases, play key roles in many aspects of neuronal development and plasticity by regulating the ECM that surrounds cells (13, 24–30); the ECM is also an important mediator of glia–neuron interactions (31). The cell-specific transcriptional regulation of Mmps could therefore have a significant impact on glia–neuron interactions and nervous system function, but remains largely unresolved. We have undertaken a genetic approach to investigate the role of glial signaling in the regulation of expression of Mmps in the *Drosophila* peripheral motor system. Through a small-scale glial-specific transgenic RNA interference (RNAi) screen for molecules that regulate the expression of the two *Drosophila* metalloproteinases (Mmp1 and Mmp2), we have identified the transmembrane ligand Delta as a prominent transcriptional regulator of Mmp1 in both central and peripheral SPG. Our genetic analysis of the *Drosophila* larval neuromuscular system indicates that a constitutive inhibitory pressure on c-Jun N-terminal kinase (JNK) signaling provided by Delta/Notch in SPG is essential for controlling the expression of Mmp1. This inhibitory signal ensures ECM and SJ integrity and appropriate ensheathment and function of motor nerve bundles. We find that when this regulatory signal is perturbed, SJs, ensheathment, and the associated barrier function are impaired and, as a result, muscle contractions and neurotransmitter release are compromised.

## Results

### Delta/Notch Signaling Regulates Mmp1 Expression in Glia.

To investigate the molecular mechanisms that regulate the expression of the *Drosophila* Mmps, we conducted a small-scale tissue-specific transgenic RNAi screen in third-instar larvae. Taking advantage of the *GAL4/UAS* expression system and the pan-glial driver *Repo-GAL4* (32, 33), we obtained *UAS*-driven RNAi lines for 14 major signal transduction pathways and cell adhesion complexes (*SI Appendix*, Table S1). Among all candidates, only pan-glial knockdown of Delta led to a significant (∼9-fold) increase in the expression of Mmp1 messenger RNA (mRNA) (*SI Appendix*, Table S1) in the central nervous system (CNS). We did not detect any changes in Mmp2 mRNA in response to any of our genetic manipulations; therefore, we did not pursue the role of Mmp2 any further. To characterize the expression pattern of Delta in glia, we used a LacZ enhancer trap line (*delta^05151^*) in late larval stages, which showed clearly that Delta is transcribed in both central and peripheral glia in the vicinity of muscle 4 (m4) (*SI Appendix*, Fig. S1 *A* and *B*). Furthermore, assessment of pan-glial Delta knockdown in peripheral nerves revealed a 50% reduction of Delta protein expression (*SI Appendix*, Fig. S1 *C* and *D*). We confirmed the induction of Mmp1 mRNA following the knockdown of Delta with a second transgenic RNAi line (*SI Appendix*, Fig. S1*E*); in addition, because loss-of-function mutant combinations of *delta* are embryonic lethal, we used a hypomorphic temperature-sensitive (ts) mutant combination of *delta* (*delta^RF^/delta^RevF10^*) and restricted it to postembryonic larval stages, and found a similar enhancement of Mmp1 mRNA levels (*SI Appendix*, Fig. S1*F*).

**Fig. 1.**
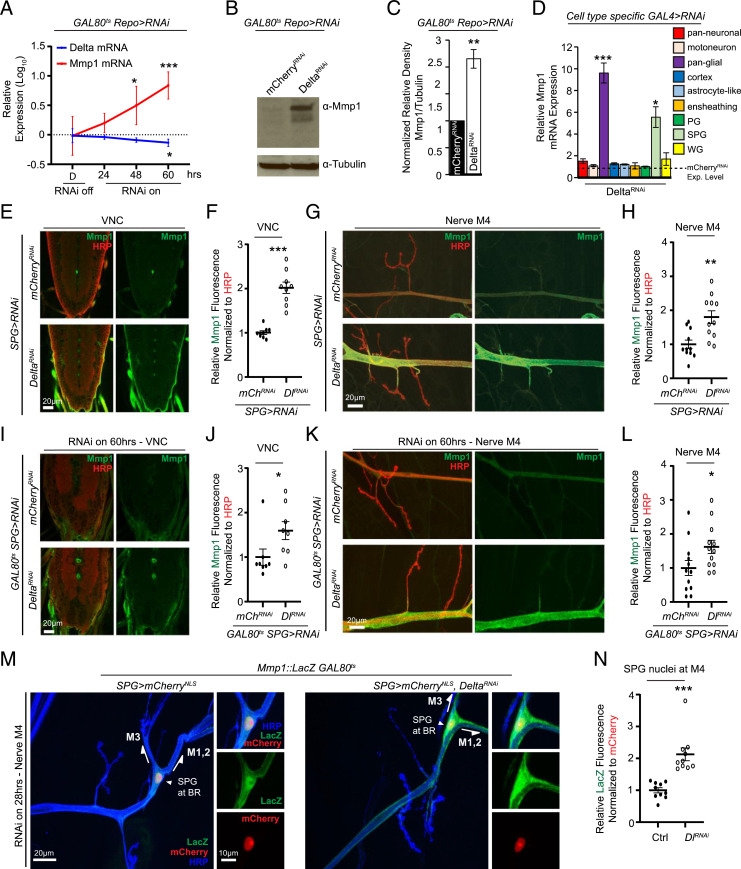
Delta negatively regulates Mmp1 in glia. (*A*) qRT-PCR analysis showing *mmp1* and *delta* mRNA expression in larval CNS of *tub-GAL80^ts^;Repo-GAL4/UAS-Delta^RNA^* relative to *tub-GAL80^ts^;Repo-GAL4/UAS-mCherry^RNAi^* (not depicted in figure) maintained at 18 °C or transferred to 29 °C for the indicated times, *n* = 5 for each genotype and condition followed by one-way ANOVA and Dunnett’s multiple-comparison test against the control for each genotype. (*B*) Western blot of the CNS of *tub-GAL80^ts^;Repo-GAL4/UAS-mCherry^RNAi^* or *UAS-Delta^RNAi^* transferred to 29 °C for 60 h and blotted for anti-Mmp1 and anti-tubulin. (*C*) Quantification of the relative density of Mmp1 signal in *B*, *n* = 3 for each genotype followed by Student’s *t* test. (*D*) qRT-PCR analysis showing relative *mmp1* mRNA expression in the CNS of *Elav-GAL4* (pan-neuronal, *n* = 5), *OK371-GAL4* (motoneuron, *n* = 5), *Repo-GAL4* (pan-glial, *n* = 5), *GMR54H02-GAL4* (cortex, *n* = 3), *alrm-GAL4* (astrocyte-like, *n* = 3), *GMR56F03-GAL4* (ensheathing, *n* = 3), *c527-GAL4* (PG, *n* = 3), *moody-GAL4* (SPG, *n* = 5), and *nrv2-GAL4* (WG, *n* = 3) crossed to *UAS-mCherry^RNAi^* or *UAS-Delta^RNAi^* followed by Student’s *t* test for each respective pair and Holm–Šídák correction for multiple comparisons. Note mCherry^RNAi^ is not depicted. (*E*) Single section of a confocal *Z* stack showing the larval VNC of *moody-GAL4/UAS-mCherry^RNAi^* or *UAS-Delta^RNAi^* stained with anti-Mmp1 (green) and anti-HRP (red). (*F*) Quantification of anti-Mmp1 fluorescence signal intensity of the VNCs in *E*, *n* = 9 for each genotype, followed by Student’s *t* test. (*G*) Maximum-intensity projection of a confocal *Z* stack of the nerve of A3M4 of the genotypes in *E* stained with anti-Mmp1 (green) and anti-HRP (red). (*H*) Quantification of anti-Mmp1 fluorescence signal intensity of the nerve in *G*, *n* = 12 for each genotype, followed by Student’s *t* test. (*I*) Single section of a confocal *Z* stack showing the larval VNC of *moody-GAL4;tub-GAL80^ts^/UAS-mCherry^RNAi^* or *UAS-Delta^RNAi^* transferred to 29 °C for 60 h and stained with anti-Mmp1 (green) and anti-HRP (red). (*J*) Quantification of anti-Mmp1 mean fluorescence signal intensity of the VNCs in *I*, *n* = 8 for each genotype followed by Student’s *t* test. (*K*) Maximum-intensity projection of a confocal *Z* stack of the nerve region of A3M4 of the genotypes in *I* stained with anti-Mmp1 (green) and anti-HRP (red). (*L*) Quantification of anti-Mmp1 mean fluorescence signal intensity of the nerve in *K*, *n* = 12 for each genotype followed by Student’s *t* test. (*M*) Maximum-intensity projection of a confocal *Z* stack of the nerve of A3M4 of *moody-GAL4,UAS-mCherry^NLS^/Mmp1::LacZ;GAL80^ts^* (control) or *moody-GAL4,UAS-mCherry^NLS^/Mmp1:LacZ;GAL80^ts^*/*UAS-Delta^RNAi^* transferred to 29 °C for 28 h and stained with anti-LacZ (green) and anti-HRP (blue). Magnified panels of M4 and the branchpoint (BR) are shown (*Right*). (*N*) Quantification of anti-LacZ fluorescence signal intensity of the nuclei in *M*, *n* = 10 for each genotype. Signal was quantified within the volume occupied by mCherry signal. Significance is determined by Student’s *t* test. *P < 0.05; **P < 0.01 and ***P < 0.001. All error bars are standard error of the mean.

The importance of Delta/Notch signaling during embryonic development (34–36), and our results with the *delta^RF^/delta^RevF10^* combination, led us to use ts expression of the inhibitor of GAL4, GAL80 (37), to restrict Delta knockdown to postembryonic stages and further assess postembryonic regulation of Mmp1. Using GAL80^ts^, temporally controlled Delta knockdown postembryonically in all glia led to an enhancement in Mmp1 mRNA expression in the CNS, and an increase in protein expression both in the CNS and PNS (Fig. 1 *A*–*C* and *SI Appendix*, Fig. S1 *G–J*). This suggests that transcriptional inhibition of Mmp1 expression by Delta persists throughout postembryonic larval stages.

To assess whether Delta is required in specific glial subtypes to regulate Mmp1 expression, and whether neurons participate in this regulation, we used different neuronal and glial drivers to knock down the expression of Delta. We first determined whether motoneurons specifically transcribe Delta by combining *delta^05151^* with *OK371-GAL4* (38) driving the expression of enhanced green fluorescent protein (eGFP) (*SI Appendix*, Fig. S1*K*). However, despite the presence of Delta transcription in motoneurons (*SI Appendix*, Fig. S1*K*), knockdown of Delta in motoneurons (*SI Appendix*, Fig. S1*L*) had no effect on Mmp1 transcript levels (Fig. 1*D*). Similarly, while our assessment of Mmp1 transcript levels indicated that the majority of Delta expression appears to be neuronal (*SI Appendix*, Fig. S1*L*), knockdown of Delta in all neurons did not show a statistically significant effect on the expression of Mmp1 (Fig. 1*D*), suggesting that the negative regulation of Mmp1 is dependent largely on a glial-initiated Delta signal. Of all the glial drivers probed, only transgenic knockdown of Delta in SPG showed a strong induction of Mmp1 mRNA compared with the same manipulation in other glial subtypes (Fig. 1*D*; see *SI Appendix*, Table S2 for GAL4 driver tissue-specific expression description). Knockdown of Delta in SPG throughout development showed a strong increase in the Mmp1 protein levels in the ventral nerve cord (VNC) (Fig. 1 *E* and *F*) and in the peripheral nerve (Fig. 1 *G* and *H*). Limiting the knockdown to postembryonic stages using *tubGAL80^ts^* was sufficient to increase the Mmp1 protein levels (Fig. 1 *I*–*L*). Approximately nine stereotypically located nuclei can be detected along the nerve innervating abdominal segment 3 (A3), M4 of each hemisegment in mature larvae; of these, three correspond to SPG, two to WG, and the rest are PG (14, 39). One SPG nucleus is easily located and visualized with the SPG driver *moody-GAL4* (40, 41), as it resides close to M4 and the branchpoint between dorsal muscles 1, 2, and 3 on the ISN (intersegmental nerve) nerve bundle (*SI Appendix*, Fig. S2*A*) (14, 39). Another easily visualized nucleus close to M4 is a WG nucleus, but it resides closer to the NMJ than the branchpoint as identified by the WG driver *nrv2-GAL4* (42) (*SI Appendix*, Fig. S2*A*). To further confirm the location of this SPG nucleus and that SPG express Delta, we combined the LacZ enhancer trap line *delta^05151^* with another SPG driver, *gliotactin* (*rl82*)*-GAL4* (9), driving the expression of eGFP, and probed for LacZ and eGFP signal overlap in the CNS and the SPG nucleus in the vicinity of M4 (*SI Appendix*, Fig. S2 *B* and *C*). Finally, to determine if Mmp1 transcription is up-regulated in this peripheral SPG nucleus following Delta knockdown, we combined a transgenic Mmp1-LacZ transcriptional reporter (43) with temporally regulated Delta-RNAi and a nuclear mCherry signal being driven by the SPG driver *moody-GAL4*. This experiment revealed an up-regulation of the LacZ signal following Delta knockdown in mCherry-positive nuclei in the vicinity of M4, indicating increased Mmp1 transcription (Fig. 1 *M* and *N*). Our findings demonstrate the presence of Delta in late stages of larval life both in neurons and glia of the CNS and along motor nerve bundles (44, 45), and identify Delta in SPG as a critical regulator of Mmp1 expression.

We next asked whether this function of Delta in SPG depends on its cognate receptor, Notch. There are three hypothetical scenarios: Delta in SPG signals to Notch in neurons, Delta in SPG *cis*-inhibits Notch in SPG receiving signal from Delta in another cell, or Delta in SPG signals to Notch in glia (either another type of glia or another SPG cell). The first scenario was ruled out, as knockdown of Notch in a nontemporal or temporal manner in all neurons, or specifically in motoneurons, had no effect on Mmp1 levels (*SI Appendix*, Fig. S3 *A* and *B*). The second scenario predicts that knockdown of Delta in SPG [removal of *cis*-inhibition (46, 47)] would lead to an enhancement in Notch signaling in SPG, and therefore activation of Notch in SPG should phenocopy knockdown of Delta in glia. However, we found that activation of Notch signaling in SPG or glia using a constitutively active form of Notch (Notch intracellular domain; Notch^ICD^) led to a mild reduction in Mmp1 expression (*SI Appendix*, Fig. S3*C*). Indeed, activation of Notch in SPG counteracts the effect of Delta knockdown in increasing Mmp1 levels (*SI Appendix*, Fig. S3*D*). Our results support the third scenario: We found that transgenic knockdown of Notch in glia led to a qualitatively similar increase in Mmp1 levels as we saw in response to knockdown of Delta (*SI Appendix*, Fig. S3 *E* and *F*). In order to determine which glial subtype is relevant for Notch-mediated Mmp1 regulation, we expressed Notch-RNAi in different peripheral glial subtypes using specific drivers. We found that, similar to the case of Delta knockdown, knockdown of Notch in SPG had the largest effect on Mmp1 expression levels (*SI Appendix*, Fig. S3*G*). These results together indicate that Mmp1 expression is under constitutive inhibitory pressure provided by Delta/Notch signaling in SPG cells.

### Delta Is Required in SPG for the Maintenance of Glial Ensheathment of Motor Nerve Bundles.

Our immunohistochemical analysis of Mmp1 expression in the PNS showed a significant increase in Mmp1 protein on the nerve (Fig. 1 *G* and *K*). To determine if the increase in Mmp1 was associated with increased proteolytic activity, we performed in situ zymography experiments following Delta knockdown in SPG cells. In this experiment, the dissected larval fillet is incubated with a highly quenched, fluorescein-labeled gelatin targeted by Mmps. Upon proteolytic digestion, the gelatin’s bright, green fluorescence is revealed. This experiment revealed that Delta knockdown in SPG cells is also associated with increased Mmp proteolytic activity (*SI Appendix*, Fig. S4).

**Fig. 2.**
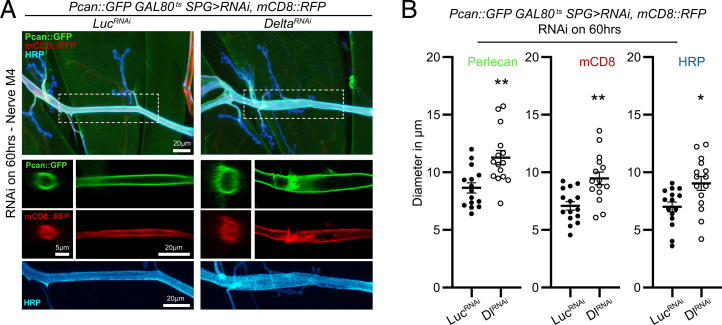
Delta knockdown in SPG remodels the glial neural lamella ECM around the nerve. (*A*, *Top*) Maximum-intensity projection of a confocal *Z* stack of the larval motor nerve at the NMJ of A3M4 of *Pcan::GFP;tub-GAL80^ts^/moody-GAL4,UAS-mCD8::RFP;UAS-Luciferase^RNAi^* or *UAS-Delta^RNAi^* transferred to 29 °C for 60 h and stained with anti-HRP (blue). (*A*, *Middle*) Orthogonal view and single section of the confocal *Z* stack from the selected area showing only the Pcan::GFP or mCD8::RFP signal. (*A*, *Bottom*) Maximum-intensity projection of a confocal *Z* stack showing only the HRP signal. (*B*) Diameter of the nerve as delineated by Pcan::GFP (*Left*), mCD8::RFP (*Center*), or HRP (*Right*) for the genotypes in *A*, *n* = 15 for each genotype followed by Student’s *t* test for each respective pair. *P < 0.05 and **P < 0.01. All error bars are standard error of the mean.

The increase in Mmp1 expression and activity also appeared to be associated with an enlarged nerve diameter (Figs. 1 *G* and *K* and 2*A*). We defined this morphological abnormality to represent a defect in the normal ensheathment of the nerve bundle. To quantify changes in nerve morphology, we used transgenic reporters for ECM components that mainly reside in the neural lamella, the proteoglycan Perlecan (Pcan::GFP) (13) and the collagen IV Viking (Vkg::GFP) (27, 48). We also combined the Perlecan reporter with *UAS*-driven expression of the membrane marker mCD8::RFP, to further assess changes in nerve morphology. In a temporally regulated manner, we knocked down Delta with *moody-GAL4* and measured nerve diameter defined by Pcan::GFP, mCD8::RFP, and horseradish peroxidase (HRP) in a region encompassing M4 in the A3 segment. This genetic manipulation revealed a significant expansion of the diameter of the nerve bundle (Fig. 2 *A* and *B*). To determine if Mmp1 is causing this expansion, we attempted overexpression of an Mmp1 transgene with the same SPG driver, but it proved to be lethal even combined with temperature-sensitive regulated expression (GAL80^ts^). Nevertheless, using milder expression conditions than for Delta-RNAi (lower temperature 27 vs. 29 °C and shorter incubation time 48 vs. 60 h), we were able to obtain viable larvae of transgenic Mmp1 overexpression with *moody-GAL4* and detect a small expansion in the Perlecan and HRP signal (*SI Appendix*, Fig. S5 *A* and *B*), suggesting that Mmp1 is causing nerve expansion. To rule out if a Delta-initiated signal in neurons can also influence nerve ensheathment, we analyzed nerve diameter defined by Pcan::GFP while knocking down Delta with the pan-neuronal driver *Elav-GAL4*. We found no changes in nerve diameter in this genetic combination, suggesting that Delta specifically in SPG is required for maintaining glial ensheathment of nerve bundles (*SI Appendix*, Fig. S5 *C* and *D*).

Knockdown of Delta in SPG led to a similar expansion of the nerve diameter defined by Vkg::GFP and HRP (*SI Appendix*, Fig. S5 *E–G*). However, in Mmp1 heterozygous larvae, knockdown of Delta did not cause any detectable change in the diameter of the bundle (*SI Appendix*, Fig. S5 *E–G*). This dominant suppression phenotype supports the critical role of Mmp1 in mediating the damaging effect of Delta knockdown.

Finally, to further confirm whether Delta in SPG is required to maintain normal nerve ensheathment, we rescued the hypomorphic ts mutant of *delta* by driving the expression of Delta in SPG with *moody-GAL4*. Restoring Delta expression in SPG in *delta* mutant larvae restored normal nerve morphology (*SI Appendix*, Fig. S5 *H* and *I*). Taken together, these data indicate that Delta signaling in SPG is essential for preserving ECM structure associated with the neural lamella and for maintaining the normal morphology of glial ensheathment of nerve bundles during postembryonic larval stages.

### Delta Is Required in SPG for the Maintenance of Barrier Function along Motor Nerve Bundles.

**Fig. 3.**
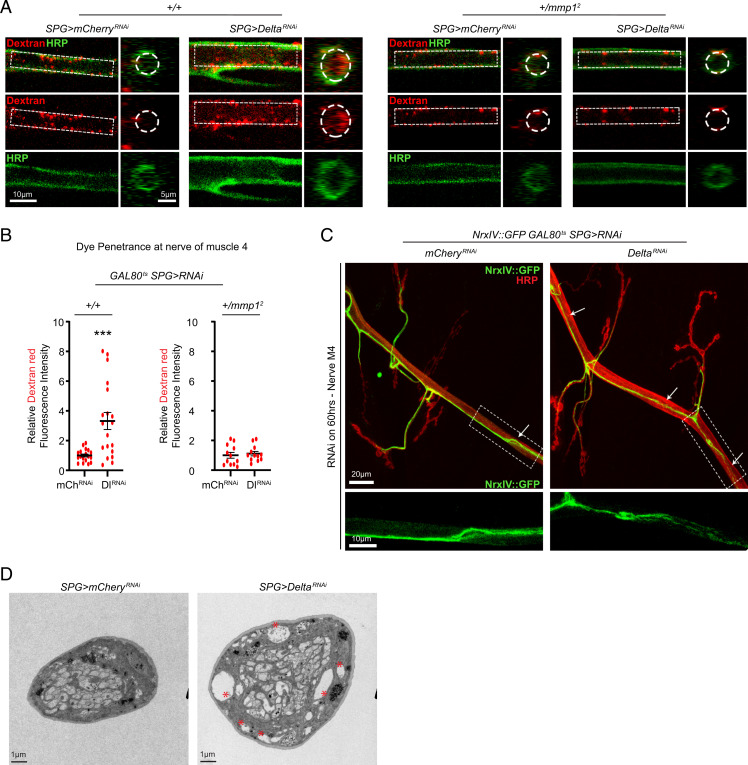
Delta knockdown in SPG disrupts barrier function. (*A*) Single section of a confocal *Z* stack showing the motor nerve at the NMJ of A3M4 of *moody-GAL4/UAS-mCherry^RNAi^* or *UAS-Delta^RNAi^* (*Left*), or *mmp1^+/2^;moody-GAL4/UAS-mCherry^RNAi^* or *UAS-Delta^RNAi^* (*Right*) following injection with dextran (red) and stained with anti-HRP (green). The dotted area shows dye penetration inside the nerve and the area chosen to assess dye intensity. (*B*) Quantification of the dextran fluorescence intensity signal in *A*, *Left*, *n* = 18 for mCherry^RNAi^ and *n* = 19 for Delta^RNAi^ or *A*, *Right*, *n* = 12 for each genotype followed by Student’s *t* test. (*C*, *Top*) Maximum-intensity projection of a confocal *Z* stack of the nerve at NMJ A3M4 of *tub-GAL80^ts^/moody-GAL4;NrxIV::GFP/UAS-mCherry^RNAi^* or *UAS-Delta^RNAi^* transferred to 29 °C for 60 h and stained with anti-HRP (red). Arrows indicate FAs. (*C*, *Bottom*) Magnification of the selected area showing severe fraying of the NrxIV::GFP signal in the Delta^RNAi^ condition compared with the mCherry^RNAi^ condition; 0.7 FA per nerve for mCherry^RNAi^, *n* = 12 vs. 3.2 FA per nerve for Delta^RNAi^, *n* = 12, Mann–Whitney *U* test, *P* = 0.02. (*D*) Ultrastructure of third-instar larval nerves of *moody-GAL4/UAS-mCherry^RNAi^* or *UAS-Delta^RNAi^*. The nerve diameter is larger in Delta knockdown, and vacuole-like structures can be detected (asterisks). ***P < 0.001. All error bars are standard error of the mean.

The changes we observed in nerve morphology have been associated with impaired glial ensheathment and SJ damage, which lead to defective barrier function (8, 11, 15). To assess if the observed glial-initiated changes in the ECM lead to increased paracellular diffusion resulting from a defective barrier, we set out to test whether transgenic knockdown of Delta in SPG was sufficient to compromise barrier function as measured by the penetration of a fluorescently labeled dye into the peripheral nerve space and the VNC (49). Delta knockdown and control larvae were injected with an amine fixable fluorescent dextran dye (*Materials and Methods*) (6), dissected, fixed, and stained with anti-HRP in nonpermeabilizing conditions so as to not disrupt the assessment of dye penetration. Both for control and knockdown conditions, the analysis of a single image of a confocal *Z* stack revealed that the nonpermeabilizing conditions did not allow the HRP signal to fully penetrate the nerve, but knockdown of Delta in SPG revealed increased penetration of the low molecular mass dye (10 kDa) both in the peripheral nerve space of a region encompassing M4 and the VNC (Fig. 3 *A* and *B* and *SI Appendix*, Fig. S6 *A* and *B*). Furthermore, our genetic analysis demonstrated that Mmp1 heterozygous larvae maintained their barrier function along motor nerves despite the knockdown of Delta with *moody-GAL4*, but not in the VNC where dye penetration was still observed (Fig. 3 *A* and *B* and *SI Appendix*, Fig. S6 *A* and *B*). This dominant suppression genetic interaction indicates that restriction of Mmp1 expression by Delta is key for maintaining the barrier function in peripheral motor nerves.

In order to determine if underlying changes in SJs could explain the deficits in barrier function, we first directly disrupted SJs by knocking down neurexin IV (NrxIV) (8, 10), a critical structural component of the SJ, to see if it recapitulated the changes in nerve morphology we observe. Knockdown of NrxIV with *moody-GAL4* led to a strong expansion in nerve diameter defined by HRP (*SI Appendix*, Fig. S6 *D* and *E*), as has been previously observed (8). In contrast to knockdown of Delta, which induces both the expansion of the nerve diameter and increase in Mmp1 protein expression, knockdown of NrxIV did not significantly increase the Mmp1 protein level in the A3M4 nerve (*SI Appendix*, Fig. S6 *F–H*). Similarly, we did not detect any changes in the mRNA levels for Mmp1 or Mmp2 using qPCR analysis (*SI Appendix*, Fig. S6*I*).

Next, we analyzed the pattern of expression of an NrxIV genomic reporter (NrxIV::GFP), which normally appears as a continuous line that follows the nerve bundle and marks the boundaries of SPG (7, 8, 10, 50). We quantified the number of frayed areas (FAs), defined by the disruption of the continuous linear NrxIV::GFP signal, in both control and temporally regulated Delta knockdown (with *moody-GAL4*) on the nerve bundle at M4 (Fig. 3*C*). The prevalence of these areas was much higher, and the loss of signal appeared more severe in response to Delta knockdown (0.7 FA per nerve for mCherry^RNAi^, *n* = 12 vs. 3.2 FA per nerve for Delta^RNAi^, *n* = 12, Mann–Whitney *U* test, *P* = 0.02). Finally, to further characterize the changes in nerve morphology in Delta-deficient SPG nerves, we performed ultrastructural analysis. This analysis revealed that knockdown of Delta with *moody-GAL4* caused the appearance of vacuole-like structures in orthogonal slices of third-instar larval nerves (Fig. 3*D*), as has been previously reported in nerves with disrupted barrier function (8). The number (1.7 vacuoles per nerve cross-section for mCherry^RNAi^, *n* = 10 vs. 4.50 vacuoles per nerve cross-section for Delta^RNAi^, *n* = 12, Mann–Whitney *U* test, *P* = 0.023) and size (0.24 µm in diameter for mCherry^RNAi^, *n* = 17 vs. 0.84 µm in diameter for Delta^RNAi^, *n* = 54, Student’s *t* test, *P* = 0.0005) of vacuole-like structures were greatly increased in Delta knockdown nerves. Delta knockdown also appeared to make some nerves too fragile for the electron microscopy fixation process (*SI Appendix*, Fig. S6*C*). Our results indicate that Delta is required in SPG to maintain NrxIV-containing SJs and barrier function in peripheral motor nerves.

### Transgenic Knockdown of Delta in SPG Alters Coordinated Muscle Contraction and Synaptic Strength at the NMJ.

Previous studies have revealed the importance of SJs and glial ensheathment for axonal signal propagation, nerve excitability, and coordinated muscle contractions (9–12). A major role of the barrier function in *Drosophila* nerves is to isolate axons from the high concentration of potassium (K^+^) in larval hemolymph (9, 10). To ascertain if the breakdown of barrier function as a result of Delta knockdown in SPG impaired coordinated muscle contractions, we imaged muscle contractions of larvae as we dissected them in low-K^+^ HL3 and immediately replaced the solution with high-K^+^ HL3 (Movie S1, mCherry^RNAi^ and Movie S2, Delta^RNAi^). These time-lapse images converted to videos revealed a 70% reduction in muscle contractile speed in Delta knockdown condition compared with control (*SI Appendix*, Fig. S7*A*). Removal of one gene copy of Mmp1 larvae was sufficient to restore normal contractile speed, indicating barrier function breakdown associated with transgenic knockdown of Delta in SPG is largely mediated by Mmp1 (*SI Appendix*, Fig. S7*B*, Movie S3, Mmp1 het mCherry^RNAi^, and Movie S4, Mmp1 het Delta^RNAi^). Interestingly, as shown previously (Fig. 3 and *SI Appendix*, Fig. S6), while heterozygosity of Mmp1 was sufficient to restore barrier function in motor nerve bundles, we could still detect dye penetration in the VNC. These results together suggest that defects in neuronal excitability and conduction, as related to muscle contraction, are largely dependent on peripheral and not central barrier function.

**Fig. 4.**
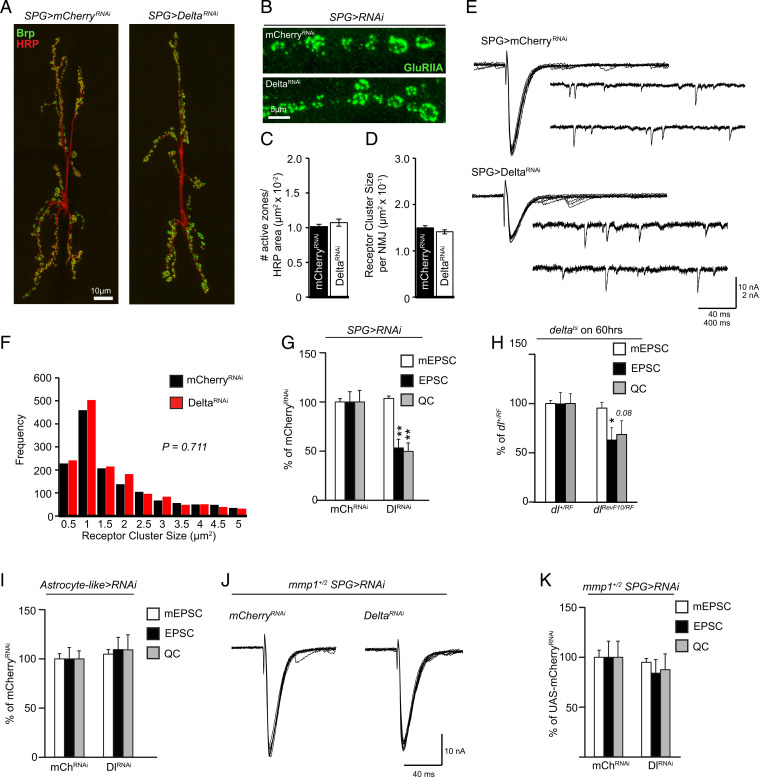
Delta regulates synaptic strength at the NMJ. (*A* and *B*) Maximum-intensity projections of the NMJ of A3M6/7 of *moody-GAL4/UAS-mCherry^RNAi^* or *UAS-Delta^RNAi^* stained with (*A*) anti-HRP (red) and anti-Brp (green) or (*B*) anti-GluRIIA (green). Images in *A* are composites of two high-magnification images. (*C*) Quantification of the number of active zones defined by anti-Brp puncta in *A* (*n* = 18 for each genotype) followed by Student’s *t* test. (*D*) Quantification of the average GluRIIA cluster size per NMJ in *B* (*n* = 18 for each genotype) followed by Student’s *t* test. (*E*) Representative traces of mEPSCs and EPSCs from *moody-GAL4/UAS-mCherry^RNAi^* or *UAS-Delta^RNAi^*. (*F*) Distribution of GluRIIA receptor cluster sizes in *D* followed by the Kolmogorov–Smirnov test. (*G*) Quantification of mEPSCs, EPSCs, and quantal content (QC) from *E*, mCherry^RNAi^ (*n* = 13) or Delta^RNAi^ (*n* = 14), followed by Student’s *t* test for each respective pair. (*H*) Quantification of mEPSCs, EPSCs, and QC from *delta^+/RF^* (*n* = 12) or *delta^RF/RevF10^* (*n* = 10) followed by Student’s *t* test for each respective pair. (*I*) Quantification of mEPSCs, EPSCs, and QC from *alrm-GAL4;UAS-mCherry^RNAi^* (*n* = 14) or *UAS-Delta^RNAi^* (*n* = 12) followed by Student’s *t* test for each respective pair. (*J*) Representative traces of EPSCs from *mmp1^+/2^;moodyGAL4/UAS-mCherry^RNAi^* or *UAS-Delta^RNAi^*. (*K*) Quantification of mEPSCs, EPSCs, and QC from *J*, mCherry^RNAi^ (*n* = 9) or Delta^RNAi^ (*n* = 7), followed by Student’s *t* test for each respective pair. *P < 0.05 and **P < 0.01. All error bars are standard error of the mean.

Glial processes at the NMJ do not appear to be in close contact with the synaptic cleft at the larval NMJ, rather the synapse is enveloped by the muscle subsynaptic reticulum (22, 23). From first- to third-instar stages, larvae undergo a period of rapid growth of their size, which requires the neuronal connections to grow homeostatically to match the increasing demand of the growing muscles, a process that involves changes in the ECM and cell–cell adhesion (51–53). Surprisingly, we found no significant defects in major indices of synaptic growth at the NMJ as a consequence of knockdown of Delta in SPG: The number of active zones (defined by Bruchpilot [Brp]-positive puncta) per NMJ (54) or the postsynaptic densities (defined by glutamate receptor subunit IIA [GluRIIA] staining) remained indistinguishable compared with control larvae (Fig. 4 *A*–*D* and *F*). These results are in line with other studies that have found intact synaptic structures in SJ-deficient nerves (9, 10). Based on these results, we expected to find the baseline synaptic transmission similarly unchanged. Indeed, the size of miniature excitatory postsynaptic currents (mEPSCs) remained unaffected when Delta was transgenically knocked down in SPG (Fig. 4 *E* and *G*). However, we found a significant decrease in the average amplitude of evoked excitatory postsynaptic currents (EPSCs) in these larvae, indicating a large decrease in quantal content (Fig. 4 *E* and *G*), similar to the reduced synaptic transmission observed in NrxIV mutants (10). In addition, we found a similar reduction in the average amplitude of EPSCs and quantal content in a ts hypomorphic mutant combination for Delta (*delta^RF^/delta^RevF10^*), further supporting the importance of Delta in the maintenance of synaptic function (Fig. 4*H*). Our findings thus far point to the critical importance of Delta specifically in SPG; in order to examine the potential role of Delta in central glia, we used an astrocytic driver (*alrm-GAL4*) to knock down Delta. This manipulation did not cause any defect in synaptic function, further highlighting the importance of Delta specifically in SPG for the maintenance of synaptic strength during larval development (Fig. 4*I*).

Finally, we asked whether Mmp1 acted downstream of partial loss of Delta/Notch signaling in the regulation of synaptic function, as it does in the maintenance of barrier function. Our electrophysiological analysis indicated that heterozygosity for Mmp1 was sufficient to fully restore normal synaptic strength, demonstrating the importance of regulation of Mmp1 activity through Delta-expressing SPG cells for glial ensheathment and establishment of quantal content at the NMJ (Fig. 4 *J* and *K*). The dominant genetic interaction between Mmp1 and Delta knockdown also provides evidence that defects in quantal content at the NMJ are not likely to be a result of breakdown in barrier function in the CNS since, as shown above (*SI Appendix*, Fig. S6), breakdown of barrier function in the CNS, as a result of knockdown of Delta, persisted despite Mmp1 heterozygosity.

### Removal of Mmp1 Specifically in SPG Protects against Loss of Delta, and Overexpression of Mmp1 in SPG Phenocopies Loss of Delta.

**Fig. 5.**
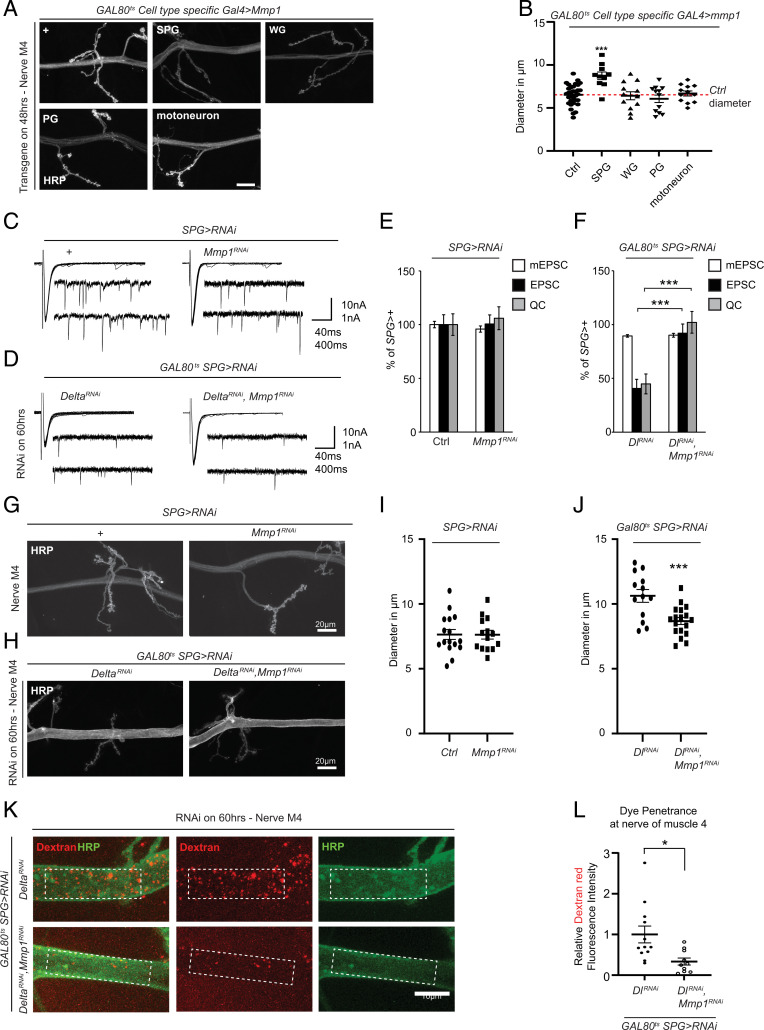
Mmp1 in SPG is responsible for defects associated with loss of Delta. (*A*) Maximum-intensity projection of a confocal *Z* stack of the larval motor nerve at the NMJ of A3M4 of *w1118* (control), *moody-GAL4* (SPG), *nrv2-GAL4* (WG), *c527-GAL4* (PG), and *BG380-GAL4* (motoneuron) crossed to *tub-Gal80ts;UAS-Mmp1* transferred to 27 °C for 48 h and stained with anti-HRP. Scale bar = 20 micron. (*B*) Diameter of the nerve as delineated by HRP for the genotypes in *A*, *n* = 33 for control, *n* = 10 for SPG, *n* = 12 for WG, *n* = 12 for PG, and *n* = 12 for motoneuron, followed by one-way ANOVA and Tukey’s test for multiple comparisons. (*C*) Representative traces of mEPSCs and EPSCs from *moody-GAL4* crossed to *w1118* (control) or *UAS-Mmp1^RNAi^*. (*D*) Representative traces of mEPSCs and EPSCs from *tub-GAL80^ts^/moody-GAL4;UAS-Delta^RNAi^* or *tub-GAL80^ts^/moody-GAL4;UAS-Delta^RNAi^/UAS-Mmp1^RNAi^* transferred to 29 °C for 60 h. (*E*) Quantification of mEPSCs, EPSCs, and QC from genotypes described in *C*, *n* = 15 for control and *n* = 13 for *UAS-Mmp1^RNAi^* followed by Student’s *t* test for each respective pair. (*F*) Quantification of mEPSCs, EPSCs, and QC from *D*, Delta^RNAi^ (*n* = 15) or Delta^RNAi^, Mmp1^RNAi^ (*n* = 17) followed by Student’s *t* test for each respective pair. (*G*) Maximum-intensity projection of a confocal *Z* stack at the larval motor nerve of A3M4 of the NMJ of *moody-GAL4* crossed to *w1118* (control) or *UAS-Mmp1^RNAi^* stained with anti-HRP. (*H*) Maximum-intensity projection of a confocal *Z* stack at the larval motor nerve at the NMJ of A3M4 of *tubGAL80^ts^/moody-GAL4;UAS-Delta^RNAi^* or *tubGAL80^ts^/moody-GAL4;UAS-Delta^RNAi^/UAS-Mmp1^RNAi^* transferred to 29 °C for 60 h and stained with anti-HRP. (*I*) Diameter of the nerve as delineated by HRP for the corresponding genotypes in *G*, *n* = 16 for control and *n* = 14 for Mmp1^RNAi^, followed by Student’s *t* test. (*J*) Diameter of the nerve as delineated by HRP for the corresponding genotypes in *H*, *n* = 13 Delta^RNAi^ and *n* = 18 for Delta^RNAi^, Mmp1^RNAi^, followed by Student’s *t* test. (*K*) Maximum-intensity projection of a confocal *Z* stack at the larval motor nerve at the NMJ of A3M4 of *tubGAL80^ts^/moody-GAL4;UAS-Delta^RNAi^* or *tubGAL80^ts^/moody-GAL4;UAS-Delta^RNAi^/UAS-Mmp1^RNAi^* transferred to 29 °C for 60 h followed by injection with dextran (red) and stained with anti-HRP (green). The dotted areas show the area chosen to assess dye intensity. (*L*) Quantification of the relative dextran fluorescence intensity signal in *K*, *n* = 12 for Delta^RNAi^ and *n* = 10 for Delta^RNAi^, Mmp1^RNAi^ followed by Student’s *t* test. *P < 0.05; **P < 0.01 and ***P < 0.001. All error bars are standard error of the mean.

Our findings thus far provide strong evidence that Mmp1 up-regulation is a key component downstream of loss of Delta in SPG; however, the specific spatial requirement of Mmp1 remains unclear. To address this, we assessed the consequence of transgenic overexpression of Mmp1 in specific glial layers as well as in motoneurons. Gal4-induced overexpression of UAS-Mmp1 in SPG did not produce any viable third-instar larvae; therefore, we took advantage of Gal4/Gal80^ts^ in order to limit the expression of SPG. We found that the maximum time of expression that produced the highest number of third-instar larvae was 48 h. We found that 48 h of Mmp1 expression in SPG, but not in WG, PG, or motoneurons, was capable of causing an expansion in the nerve diameter reminiscent of the phenotype associated with knockdown of Delta in SPG (Fig. 5 *A* and *B*). This prompted us to test whether specific knockdown of Mmp1 in SPG was sufficient to restore barrier function and synaptic activity when Delta is knocked down. We first tested the ability of a transgenic UAS-Mmp1-RNAi (Mmp1^RNAi^) to reduce the increase in Mmp1 at the NMJ when Delta is knocked down. Our immunohistochemical analysis suggested that Mmp1 RNAi can effectively reduce Mmp1 levels in the motor nerve (*SI Appendix*, Fig. S8). Interestingly, knockdown of Mmp1 in SPG did not produce any detectable defects in baseline electrophysiological properties or the normal nerve ensheathment (Fig. 5 *C*, *E*, *G*, and *I*). However, when Mmp1 was knocked down at the same time as Delta-RNAi, the electrophysiological defects associated with Delta knockdown were fully rescued (Fig. 5 *D* and *F*). Similarly, the abnormal nerve expansion associated with Delta knockdown was also significantly reduced as a result of Mmp1 knockdown. Finally, we found that abnormal dye penetration that is normally caused by Delta knockdown was significantly reduced when Mmp1 was targeted in SPG (Fig. 5 *K* and *L*). These results together indicate that Mmp1 up-regulation in SPG, rather than in other glial subtypes or motoneurons, is responsible for defects in the motor nerve barrier function and synaptic activity at the NMJ as a result of Delta/Notch signaling disruption.

### Mmp1 Regulation by Delta/Notch Is JNK-Dependent.

Our findings suggest that Delta/Notch signaling in SPG restricts Mmp1 expression, which is critical for maintaining glial ensheathment and ensuring appropriate neurotransmitter release at the NMJ. How does Delta/Notch regulate Mmp1 expression? Mmp1 is a known transcriptional target of JNK signaling (43). Therefore, we asked whether Delta/Notch controls Mmp1 transcription by regulating JNK signaling, and set out to monitor the signal associated with the Puc-LacZ insert in SPG and WG (55). This chromosomal insertion is a LacZ enhancer trap that reports changes in transcription of *puckered* (*puc*), a well-characterized target of JNK signaling (56). Indeed, we found that acute knockdown of Delta in glia using *Repo-GAL4* led to JNK activation (measured by the increase in LacZ signal) only in SPG nuclei but not in WG nuclei (*SI Appendix*, Fig. S9 *A–C*). We also used a genomically inserted JNK transcriptional reporter (TRE-GFP), which reflects JNK activation efficiently (57). Using TRE-GFP, we found a similar increase in JNK activation in SPG nuclei and cytoplasm in response to temporal knockdown of Delta in SPG with *moody-GAL4* (*SI Appendix*, Fig. S9 *D* and *E*). Finally, we asked whether knockdown of Notch in SPG is capable of producing a detectable change in JNK signaling. Consistent with a Delta/Notch interaction, we found that knockdown of Notch in SPG caused a similar increase in JNK activity in SPG nuclei (*SI Appendix*, Fig. S9 *F* and *G*).

**Fig. 6.**
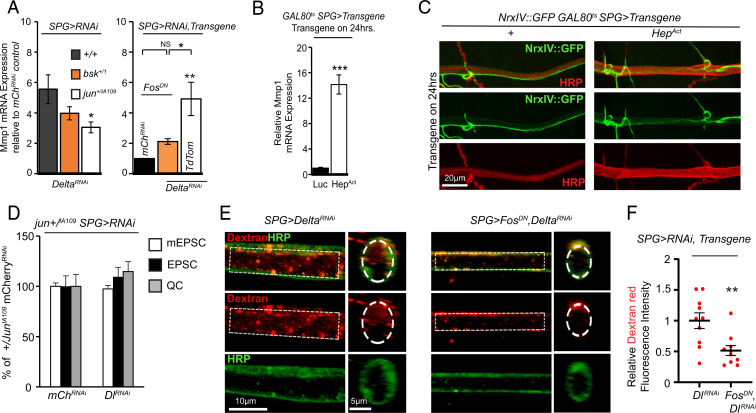
Negative regulation of Mmp1 by Delta is JNK-dependent. (*A*, *Left*) Relative *mmp1* mRNA expression in the CNS *moody-GAL4/UAS-mCherry^RNAi^* or *UAS-Delta^RNAi^*, *bsk^+/1^;moody-GAL4/UAS-mCherry^RNAi^* or *UAS-Delta^RNAi^*, or *jun^+/IA109^;moody-GAL4/UAS-mCherry^RNAi^* or *UAS-Delta^RNAi^* followed by a Kruskal–Wallis test for multiple comparisons, *n* = 5, *n* = 5, and *n* = 6, respectively, for each genotype pair. Note that levels are relative to mCherry^RNAi^ control, but genotype is not depicted. (*A*, *Right*) Relative *mmp1* mRNA expression in the CNS of *UAS-Fos^DN^;moody-GAL4/UAS-mCherry^RNAi^*, *UAS-Fos^DN^;moody-GAL4/UAS-Delta^RNAi^*, or *UAS-TdTomato;moody-GAL4/UAS-Delta^RNAi^*, *n* = 5 for each genotype followed by a one-way ANOVA with a Tukey’s test for multiple comparisons. NS, not significant. (*B*) Relative *mmp1* mRNA expression in the CNS of *tub-GAL80^ts^;UAS-Luciferase/moody-GAL4* or *UAS-Hep^Act^/tub-GAL80^ts^;moody-GAL4*, transferred to 29 °C for 24 h, *n* = 3 for each genotype followed by Student’s *t* test. (*C*) Selection of a maximum-intensity projection of a confocal *Z* stack of the nerve at NMJ A3M4 of *tub-GAL80^ts^;NrxIV::GFP/moody-GAL4* or *tub-GAL80^ts^/UAS-Hep^Act^;NrxIV::GFP/moody-GAL4* transferred to 29 °C for 24 h and stained with anti-HRP. A zoomed-out image of the same NMJ is shown in *SI Appendix*, Fig. S9*I*. (*D*) Quantification of mEPSCs, EPSCs, and QC from *jun^+/IA109^;moody-GAL4/UAS-mCherry^RNAi^ or UAS-Delta^RNAi^*, *n* = 12 for mCherry^RNAi^ and *n* = 13 for Delta^RNAi^ followed by Student’s *t* test for each respective pair. EPSC traces are shown in *SI Appendix*, Fig. S9*J*. (*E*) Single section of a confocal *Z* stack or orthogonal view showing axons at NMJ A3M4 of *moody-GAL4/UAS-Delta^RNAi^* or *UAS-Fos^DN^;moody-GAL4/UAS-Delta^RNAi^* following injection with dextran (red) and stained with anti-HRP (green). (*F*) Quantification of the relative dextran fluorescence intensity in *E*, *n* = 10 for each genotype followed by Student’s *t* test. *P < 0.05; **P < 0.01 and ***P < 0.001. All error bars are standard error of the mean.

We then tested the functional relevance of JNK signaling in mediating the increase of Mmp1 transcription as a result of Delta knockdown in SPG. Limiting JNK signaling by removing one gene copy of the kinase *basket* (*bsk*) or the transcription factor subunit *jun* was sufficient to reduce the increase in Mmp1 levels significantly when Delta was knocked down (Fig. 6*A*). We were also capable of reducing the increase in Mmp1 as a result of knockdown of Delta by coexpressing a dominant-negative (DN) transgene of another transcription factor subunit, *fos*, specifically in SPG (Fig. 6*A*). Furthermore, we tested whether direct activation of JNK signaling in SPG could lead to a transcriptional up-regulation of Mmp1. Genetic activation of JNK signaling via its upstream activator *hemipterous* (Hep^Act^) (58) led to lethality; however, we were able to conduct this experiment using temporally regulated expression with *moody-GAL4* and found that only 24 h of overexpression of Hep^Act^ was sufficient to enhance Mmp1 levels by more than 10-fold (Fig. 6*B*). These results together indicate that activation of JNK signaling in SPG is critical for transcription of Mmp1 as a result of genetic knockdown of Delta.

Based on our data thus far, we would predict that activation of JNK in SPG should compromise barrier function. We therefore examined the consequence of JNK activation in SPG for the integrity of barrier function by assessing the pattern of expression of NrxIV in nerve bundles. In larvae that had increased JNK activity (Hep^Act^ overexpressors), we found changes in NrxIV pattern expression and nerve diameter. While we could detect FAs of NrxIV signal in both control and Hep^Act^, the prevalence of these areas was much higher and more severe in larvae expressing Hep^Act^ (Fig. 6*C* and *SI Appendix*, Fig. S9*I*; 0.8 FA per nerve for control, *n* = 16 vs. 1.9 FA per nerve for Hep^Act^, *n* = 18, Mann–Whitney *U* test, *P* = 0.0024). Similar to the effect of Delta knockdown, Hep^Act^ overexpression also caused nerve expansion (*SI Appendix*, Fig. S9*H*), indicating that increased JNK signaling in SPG disrupts SJs in peripheral nerves.

Based on these findings, we set out to test the idea that limiting JNK signaling could counter the synaptic and barrier defects associated with glial knockdown of Delta. Our electrophysiological analysis in heterozygous *jun* mutants supported this idea. We found that removal of one gene copy of *jun* (*jun^+/IA109^*) protected synaptic function despite knockdown of Delta in SPG (Fig. 6*D* and *SI Appendix*, Fig. S9*J*), which normally leads to a significant reduction in EPSCs and quantal content (Fig. 4). This dominant suppression, reminiscent of dominant suppression by Mmp1, suggests strong genetic interaction and functional relevance between Delta/Notch signaling and JNK activation in the control of neurotransmitter release at the NMJ. Finally, we found that limiting JNK signaling in SPG alone was sufficient to oppose the effect of Delta knockdown: Coexpressing the DN form of Fos along with Delta^RNAi^ specifically in SPG restored barrier function as monitored in motor nerves and partially in the VNC (Fig. 6 *E* and *F* and *SI Appendix*, Fig. S9 *K* and *L*). These results together provide compelling evidence that Delta/Notch signaling in SPG exerts a constitutive inhibition of Mmp1 transcription by keeping JNK signaling in check, thereby preserving peripheral glial ensheathment and maintaining synaptic strength at the NMJ.

## Discussion

### Delta/Notch Signaling in SPG Inhibits JNK Activity Limiting Mmp1 Expression.

Previous reports have revealed the complex interaction between Notch and JNK signaling pathways in both vertebrates and invertebrates. Evidence suggests that Notch signaling can both inhibit and promote JNK signaling in a context-dependent manner. During development of the *Drosophila* embryo, a noncanonical activity of Notch inhibits JNK signaling in the patterning of the dorsal epidermis. Indeed, Notch mutants can rescue the patterning defects created by reduced JNK activity (59). Additionally, in cell-culture experiments, the intracellular domain of Notch restricts JNK signaling by inhibiting the activation of the c-Jun transcription factor or by directly associating with JNK-interacting protein 1 (60, 61). Conversely, in *Drosophila* models of tumorigenesis, a noncanonical activity of Notch in the eye in combination with Mef2 or Src has been shown to promote JNK signaling and Mmp1 expression (62, 63).

Our findings further extend the current body of knowledge by showing that Delta/Notch signaling in SPG is required to restrict JNK activity and Mmp1 expression. Our knockdown profiling of Delta or Notch in motoneurons or neurons in a constitutive or temporally controlled fashion excluded neuronal involvement in this signaling. Consistently, only knockdown of Delta or Notch in SPG altered Mmp1 transcript levels. Furthermore, activation of Notch (using Notch^ICD^) in glia or SPG alone suppressed Mmp1 levels. This result rules out the possibility that the enhancement in Mmp1 as a result of Delta knockdown is caused by an increase in Notch signaling in glia due to removal of *cis*-inhibition of Delta on Notch (34, 46, 47). These experiments, in combination with our assessment of Mmp1 or JNK in vivo reporters in SPG nuclei along peripheral motor nerve bundles, suggest that a bidirectional glia-to-glia Delta/Notch signaling appears to be controlling Mmp1 transcription via JNK signaling (35, 64). In particular, our transgenic RNAi rescue experiments indicate that limiting Mmp1 only in SPG is sufficient to significantly rescue defects associated with Delta knockdown, thus suggesting that the relevant source of Mmp1 is indeed SPG. Our results together support a model in which Delta/Notch signaling in SPG provides a constitutive negative inhibition of JNK; loss of this negative inhibition leads to an increase in JNK activity, resulting in an enhancement of Mmp1 expression in SPG. At this point, we cannot unequivocally determine whether the Delta/Notch signaling is autocellular or intercellular in SPG; further experiments are required to explore these possibilities.

### Mmp1 and the Regulation of the ECM.

Our use of in situ zymography assays and glial ECM reporters for heparan sulfate proteoglycan (*Perlecan*) and collagen IV (*Viking*) revealed increased proteolytic activity along with changes in glial ECM morphology as a result of Delta knockdown. However, we did not observe any apparent changes in the expression of these ECM markers, suggesting they are unlikely to be direct targets of proteolytic processing by Mmp1. This is consistent with what has been reported for invading tracheal branches into *Drosophila* flight muscles, where neither Perlecan nor Viking expression levels were significantly altered as direct targets of Mmp1 (65). Nevertheless, Sauerwald and colleagues (65) showed that catalytic activity of Mmp1 is required for normal tracheal invasion and remodeling of Viking-containing ECM networks, most likely through its interaction with other ECM components. Therefore, it is conceivable that the changes in the distribution of Perlecan and Viking in motor nerve bundles we observe, as a consequence of Delta knockdown, are the result of proteolytic interaction of Mmp1 with other components of the ECM. Other potential targets for proteolytic processing that could mediate Mmp1-dependent changes are *Drosophila* laminins or integrins, given their roles in glial ensheathment or in collagen-containing complexes (13, 16, 50).

In the mammalian system, several examples exist of Mmp-mediated impairment of tight junctions of the blood–brain barrier (BBB) (66), the mammalian counterpart of septate junctions in the *Drosophila* BBB and blood–nerve barrier. In many of these examples, the result was perturbation of the function of the BBB. For instance, experimental evidence indicates that abnormally enhanced Mmp activity can lead to defects in BBB function by disrupting tight junction and basement membrane proteins in Alzheimer’s disease mouse models (67). In vertebrate models of ischemia, increased Mmp activity has been shown to degrade tight junction proteins, resulting in a breach in the BBB (68, 69). Our results combined with mammalian published data suggest that up-regulated Mmp activity on the ECM has the ability to disturb the occluding junctions that are required to maintain barrier function.

Our findings suggest that, while gain of function of Mmp1 in SPG is detrimental to the integrity of the BBB, its transgenic knockdown in SPG leads to no abnormalities. This is consistent with recent results by Kanda et al. showing that Mmp2 rather than Mmp1 is critical for the proper establishment of the BBB during development (70). On the other hand, we find that Delta/Notch signaling as a critical process in the maintenance of the BBB does not influence Mmp2 but rather controls Mmp1 activity. Therefore, it appears that at different developmental stages, distinct programs have to work in concert to establish and maintain barrier function in the nervous system.

### Delta/Notch Signaling and Implications for Disease.

In human patients, mutations in Notch3 are associated with the pathogenesis of cerebral autosomal dominant arteriopathy with subcortical infarcts and leukoencephalopathy, a condition that leads to breakdown of the BBB and is associated with dementia (71). In addition, in cultured brain endothelial cells, it was found that impaired Notch signaling led to dysfunction of the BBB and increased the permeability of macromolecules, which was worsened by inflammatory conditions (72). We speculate that the Delta/Notch signaling role in maintaining barrier function and regulating occluding junctions may be part of a fundamental and conserved mechanism.

Surprisingly little is known about the regulation of Mmps outside of models of injury or inflammation in both mammals and *Drosophila* (43, 62, 63, 66, 73–75). The promoter sequences of Mmp family members are remarkably similar, particularly in the presence of JNK transcription factor AP-1 (Jun, fos) binding sites (76), a common stress/inflammation–activated pathway (77). In humans, at least a quarter of the 24 MMPs have AP-1 binding sites in their promoter sequence (76). Of these, several have been found to be abnormally expressed, perhaps as targets of inflammatory cues, in neurodegenerative diseases and associated with BBB defects (78). The discovery of the role of Delta/Notch signaling in the regulation of Mmp activity opens up a potential new avenue for novel therapeutic targets aimed at countering disease-induced damage in barrier function in the nervous system.

## Materials and Methods

### *Drosophila* Genetics.

Standard fly husbandry was performed. See *SI Appendix*, *Methods* and Table S4 for a comprehensive list of fly stocks.

### Immunohistochemistry and Imaging.

Wandering third-instar larvae were harvested for immunostaining followed by confocal microscopy. Standard techniques were used. Details are described in *SI Appendix*.

### Electrophysiology.

A standard two-electrode voltage-clamp technique was used on muscle 6 in the third abdominal segment of wandering third-instar larvae. Details are described in *SI Appendix*.

### Statistical Analysis.

All statistical analyses were performed using GraphPad Prism software or RStudio.

## Supplementary Material

Supplementary File

Supplementary File

Supplementary File

Supplementary File

Supplementary File

## Data Availability

All study data are included in the article and/or supporting information.
