# Supplementary material for: Delta/Notch signaling in glia maintains motor nerve barrier function and synaptic transmission by controlling matrix metalloproteinase expression
Source: Proc Natl Acad Sci U S A. 2022 Aug 15;119(34):e2110097119. doi: 10.1073/pnas.2110097119 (PMC9407389; doi:10.1073/pnas.2110097119)
Supplement: Supplementary File [file pnas.2110097119.sapp.pdf]

Supporting Information

Supplementary Details of Methods

Supplementary Figure S1, related to Figure 1

Supplementary Figure S2, related to Figure 1

Supplementary Figure S3, related to Figure 1

Supplementary Figure S4, related to Figure 2

Supplementary Figure S5, related to Figure 2

Supplementary Figure S6, related to Figure 3

Supplementary Figure S7, related to Figure 4

Supplementary Figure S8, related to Figure 5

Supplementary Figure S9, related to Figure 6

Table S1 Screen

Table S2 GAL4 Driver information

Table S3 Electrophysiology Data

Table S4 Fly Stocks

Table S5 Primers

Table S6 Antibodies

Movie S1 description

Movie S2 description

Movie S3 description

Movie S4 description

SUPPLEMENTAL DESCRIPTION OF METHODS

Experimental Model and Subject Details

All experiments were performed in equal number of female and male Drosophila melanogaster larvae according to standard procedures unless the genotype did not allow for this (transgene or gene investigated on the X chromosome) in which case only females were used.

Methods

Fly Genetics

Flies and larval crosses were maintained at 25°C on a 12-hour day/night cycle on standard molasses/yeast based medium following standard protocols. (see method details for recipe). For *GAL80*^{ts} and *delta*^{RF} experiments, flies were mated for 48hrs then transferred to a new vial and allowed to egg lay for 24h at 18ºC. Adults were cleared and larvae were incubated for 60hrs at 18°C and 60hrs at 29°C for the 60hrs *UAS/RF* Delta RNAi/mutation expression condition, for 72hrs at 18°C and 48hrs at 29ºC for the 48hrs Delta RNAi expression condition, for 120hrs at 18°C and 24hrs at 29°C for the 24hrs Delta RNAi expression condition, and for 96hrs at 18°C and 48hrs at 27ºC for the 48hrs Mmp1 expression condition. For *GAL80*^{ts} experiments in Figure 6 and S9C-H, mating and egg lay were performed at 25°C, but otherwise followed the 60hrs Delta RNAi expression protocol. Only wandering third instar larvae were collected for analysis. See Table S4 for a comprehensive list of fly stocks used.

Standard molasses-yeast fly food recipe

Flies were maintained on standard molasses/yeast food. Recipe as follows: 1 l distilled water, 13.8 g agar, 22 g molasses, 80 g malt extract, 18 g Brewer's yeast, 80 g corn flour, 10 g soy flour, 6.25 mL propionic acid, 2 g methyl-p-benzoate in EtOH.

Quantitative PCR

Total RNA was extracted from 6-7 wandering third instar larval female/male dissected brains per n using

the High Pure RNA isolation kit (Roche) according to the manufacturer's instructions. cDNA was prepared from total RNA with the SensiFAST cDNA Synthesis Kit (Bioline). mRNA expression was measured by quantitative real-time PCR (qPCR) with SensiFAST SYBR No-ROX Kit (Bioline) on a BioRad CFX96 thermocycler. Reference gene was Rpl32 and MIQE guidelines were followed. Refer to See Table S5 for primer sequences.

Western Blot

Wandering third instar larvae were dissected as previously described. Briefly, third instar larvae were dissected in cold HL3 and the CNS were collected. 10 CNS were collected per biological replicate for a total of 3 biological replicates. Western blot was performed according to standard protocols with the antibodies outlined in the Table S6.

Immunostaining

General Staining protocol. Wandering third instar larvae were dissected as previously described. Briefly, third instar larvae were dissected in cold HL3 and fixed with 4% Paraformaldehyde in PBS (Phosphate buffered saline) for 10 min. For figure S9C and S9D, Bouin's solution was used instead of PFA. Larval fillets were then washed with PBS three times, permeabilized and blocked with 5% Normal Goat Serum (NGS) in PBT (PBS with 0.1% Triton X-100) for 1hr and placed in primary antibody overnight at 4°C. The larvae were then washed three times for 15min in PBT, placed in secondary antibody for 2 hrs, washed three times for 15min with PBT and mounted in Vectashield (Vector labs). For a list of antibodies and dilutions used see Table S6.

Delta staining. All the steps were the same as for the general staining protocol except for the following modifications: Dissected larvae were fixed with 4% Paraformaldehyde in PBS for 10 min. The Paraformaldehyde was then replaced with 100% methanol for 5 min. The 100% methanol was replaced with 50% methanol in PBS for 2 min. The 50% methanol in PBS was replaced with PBS for 5 min and subsequent steps were the same as for the general protocol.

Mmp1 staining. All the steps were the same as for the general staining protocol except for the following modifications: All washes were in PBS only.

GluRIIA staining. All the steps were the same as for the general staining protocol except for the following modifications: Third instar larvae were fixed with Bouin's solution for 10 min. For GluRIIA cluster size, briefly, FIJI was used to threshold the images at the same level and particle sizes between 0.35-5μm were analyzed with the Analyze particle function.

Electrophysiology

Wandering third instar larvae were dissected in cold HL3 solution without Ca2+ following standard protocol. The spontaneous (mEPSC) and evoked (EPSC) membrane currents were recorded from muscle 6 in abdominal segment A3 with standard two-electrode voltage-clamp technique (1). All recordings were performed at room temperature in HL3 solution containing 0.5mM Ca2+ unless otherwise indicated. The current recordings were collected with AxoClamp2B, AxoClamp2A, and AxoClamp900A amplifiers (Molecular Devices Inc.) using Clampex 9.2 (AxoClamp2B and AxoClamp2A) and 10.4 (AxoClamp900A) software (Molecular Devices Inc.). Nerve stimulation was delivered through a suction electrode, which held the cut nerve terminal cord. In all voltage clamp recordings, muscles were held at -80 mV. All records were subjected to 1 kHz low-pass filtering during acquisition. For each NMJ, mEPSC (3 minutes continuous recording) and EPSC (40 pulses, 0.5 Hz or 40 pulses, 1Hz) amplitudes were measured using Mini Analysis 6.0.3 or 6.0.7 software (Synaptosoft) or an internally developed software and verified by eye. QC was calculated by dividing the mean EPSC amplitude by mean mEPSC amplitude. The recording traces were generated with Origin 7.5 (Origin Lab), Prism 8 (Graphpad) software, or Python (Python Software Foundation). Details of the recordings can be found in Table S3.

In situ zymography

Wandering third instar larvae were dissected and incubated in 1X reaction buffer (Molecular Probes recipe for DQ-gelatin) containing 400µg/mL of DQ-gelatin for 30min at room temperature. Preparations were rinsed 3X in PBS and then fixed for 10min in 4% PFA at room temperature. Preparations were rinsed again 3X in PBS, and incubated 2hrs with anti-HRP in PBS at room temperature. Preparations were rinsed again 3X in PBS and mounted in Vectashield.

Electron microscopy

Wandering third instar larvae were dissected in HL3 and fixed in 2% PFA and 2% glutaraldehyde in phosphate buffer at room temperature for 2 h. The larvae were then removed from the plastic support and fixed in the same solution at 4°C overnight. The samples were then sent to the Electron Microscope Lab at the University of California, Berkeley, for processing and imaging. Images were taken on FEI Tecnai 12 transmission electron microscope.

Dye Permeability Assay

Wandering third instar larvae were immobilized on tape and injected in the posterior body with a machine-drawn capillary needle filled with Dextran 10kDa (2.5mM final concentration in water). Larvae were injected until the entire body turned the color of the dye (~100nL of Dextran). Larvae were then rolled in halocarbon oil for recovery and placed on molasses plates after the excess oil was wiped off. 15 min post injection, larvae were dissected as in the general staining protocol. Dissected larvae were then washed 3 times with PBS and fixed with 4% Paraformaldehyde in PBS for 10 min. Dissected larvae were washed 3 times with PBS and incubated with 5% NGS and anti-HRP (Jackson ImmunoResearch) in PBS for 30 min. Dissected larvae were washed 3 times with PBS, mounted in Vectashield, and immediately imaged. Z-stacks of the VNC and the nerve at NMJ (M4) were taken and the fluorescent Dextran signal was quantified (FIJI) using the single frame image that showed the largest structure of the VNC or NMJ on the same plane. For Figure S9G, analysis was performed in Maximum Intensity Projection of 30 Z-stack slices. Regions of interest (ROI) were manually selected using the HRP signal. Mean Gray Value of

the background was subtracted from the Mean Gray Value of the ROI. Surface areas analyzed between control and experiment were not significantly different as determined by Student's T-test.

Confocal imaging and image analysis

Brain and NMJ images were captured using Zeiss LSM 700 and LSM 780 confocal microscopes with 40x/1.4 and 40x/1.3-63x/1.4 oil objectives respectively with Zen software. Image analysis was performed using FIJI software or Imaris image analysis software (Bitplane). Figures were made in Adobe Illustrator and Photoshop (CS6).

Statistical Analysis

All statistical analyses were performed using GraphPad Prism software or RStudio (RStudio). Further statistical details for each experiment can be found in the corresponding figure legend. Mean ± s.e.m is shown. Asterisk P-values are as follows: *≤0.05, **≤0.01, ***≤0.001.

Time Lapse Images of Dissected larvae

Briefly, third instar larvae were dissected in cold HL3 and then transferred under a Zeiss SterREO Discovery V20 microscope equipped with a camera. The HL3 was then switched to HL3 high in potassium (high K_+) HL3 saline, adjusted to maintain osmolarity (in mM): NaCl 40, KCl 90, $CaCl_2$ 1.5, $MgCl_2$ 20, $NaHCO_3$ 10, trehalose 5, HEPES 5, sucrose 5. The larvae were recorded for 2 min at ~2.3 frames per second with the SPOT 5.0 software. The "export video" function of SPOT was used to generate a 10-11 seconds video from the time lapse images. To analyse the muscle contractile speed, the time lapse image series was used with the FIJI software plugin TrackMate v5.0.2 (2). The first 30 images of the time-lapse image series were excluded from the analysis as it showed the transition from low K_+ to high K_+ HL3. The plugin detected an average of 30 particles per larvae, and the mean speed of the 30 particles was averaged to obtain an average contractile speed for the entire larvae.

Figure S1

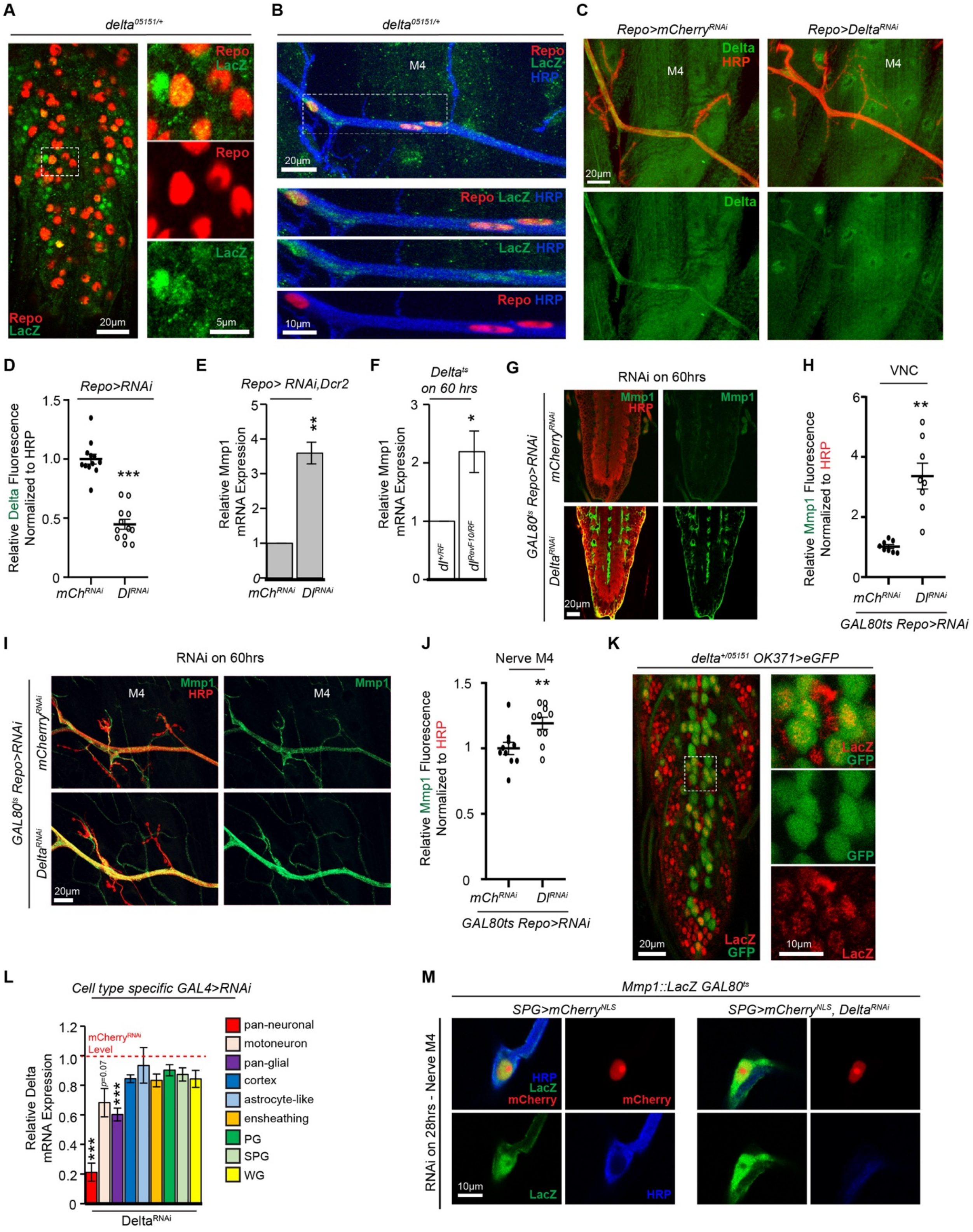
A
delta05151/+
Repo
LacZ
20µm
5µm
B
M4
HRP
20µm
10µm
C
Repo>mCherryRNAi
Repo>DeltaRNAi
Delta
D
Repo>RNAi
Relative Delta Fluorescence Normalized to HRP
mChRNAi
DlRNAi
E
Repo> RNAi,Dcr2
Relative Mmp1 mRNA Expression
F
Deltats on 60 hrs
dl+/RF
dlRevF10/RF
G
RNAi on 60hrs
GAL80ts Repo>RNAi
Mmp1
H
VNC
Relative Mmp1 Fluorescence Normalized to HRP
GAL80ts Repo>RNAi
I
J
Nerve M4
K
delta+/05151 OK371>eGFP
GFP
L
Cell type specific GAL4>RNAi
mCherryRNAi Level
Relative Delta mRNA Expression
p=0.07
DeltaRNAi
pan-neuronal
motoneuron
pan-glial
cortex
astrocyte-like
ensheathing
PG
SPG
WG
M
Mmp1::LacZ GAL80ts
SPG>mCherryNLS
SPG>mCherryNLS, DeltaRNAi
RNAi on 28hrs - Nerve M4
mCherry

Figure S1. Delta is expressed in SPG and knockdown induces Mmp1 expression. (A) Single section of a confocal Z-stack of immunohistochemical staining showing the VNC of *delta*$^{+/05151}$ stained with anti-Repo (red) and anti-LacZ (green) along with a magnification of the selected area showing a Repo and LacZ positive cell. (B) Top. Maximum intensity projection of a confocal Z-stack of the nerve at the NMJ of A3M4 of *delta*$^{+/05151}$ stained with anti-Repo (red), anti-LacZ (green), and anti-HRP (blue). Bottom. Magnification of selected area showing Repo and LacZ positive cells. (C) Maximum intensity projection of a confocal Z-stack of the nerve at the NMJ of A3M4 of *Repo-GAL4/UAS-mCherry*^{RNAi} or *UAS-Delta*^{RNAi} stained with anti-Delta (green) and anti-HRP (red). (D) Quantification of Delta fluorescence intensity of the nerve in (C) n=12 for each genotype followed by Student's T-test. (E) RT-qPCR analysis showing *mmp1* mRNA expression in the CNS of *Repo-GAL4;UAS-Dcr2;Repo-GAL4/UAS-mCherry*^{RNAi} or *UAS-Delta*^{RNAi}*,* n=3 for each genotype followed by Student's T-test. (F) RT-qPCR analysis showing relative *mmp1* mRNA expression in the CNS of *delta*$^{+/RF}$ or *delta*$^{RF/RevF10}$ transferred to 29ºC for 60hrs followed by Student's T-test n=3 for each genotype. (G) Single section of a confocal Z-stack showing the larval ventral nerve cord (VNC) of *tub-GAL80*^{ts}*;Repo-GAL4/UAS-mCherry*^{RNAi} or *UAS-Delta*^{RNAi} transferred to 29ºC for 60hrs and stained with anti-Mmp1 (green) and anti-HRP (red). (H) Quantification of Mmp1 fluorescence intensity of the VNCs in (G), n=8 for each genotype. (I) Maximum intensity projection of a confocal Z-stack of the NMJ region of A3M4 of the genotypes in (G) stained with anti-Mmp1 (green) and anti-HRP (red). (J) Quantification of Mmp1 fluorescence intensity of the nerve in (I), n=10 for each genotype, followed by Student's T-test. (K) Single section of a confocal Z-stack of immunohistochemical staining showing the VNC of *OK371-GAL4*;*UAS-eGFP/ delta*^{05151} stained with anti-LacZ (red) along with a magnification of the selected area showing GFP and LacZ positive cells. (L) RT-qPCR analysis showing relative *delta* mRNA expression in the CNS of *Elav-GAL4* (pan-neuronal, n=5), *OK371-GAL4* (motoneuron, n=5), *Repo-GAL4* (pan-glial, n=5), *GMR54H02-GAL4* (cortex, n=3), *alrm-GAL4* (astrocyte-like, n=3), *GMR56F03-GAL4* (ensheathing, n=3)*, c527-GAL4* (PG, n=3), *moody-GAL4* (SPG, n=5), *nrv2-GAL4* (WG, n=3) crossed to *UAS-mCherry*^{RNAi} or *UAS-Delta*^{RNAi} followed by Student's T-test for each respective pair and Holm-Šídák correction for multiple comparisons. Note mCherry^{RNAi} is not depicted. (M) Single slice of a confocal Z-stack of the nucleus in the vicinity of M4 of *moody-GAL4,UAS-mCherry*^{NLS}*/Mmp1::LacZ;GAL80*^{ts} or *moody-GAL4,UAS-mCherry*^{NLS}*/Mmp1::LacZ;GAL80*^{ts}*/UAS-Delta*^{RNAi} transferred to 29°C for 28hrs and stained with anti-LacZ (green) and anti-HRP (blue). Maximum intensity projections of the corresponding images are shown in main Figure 1.

Figure S2

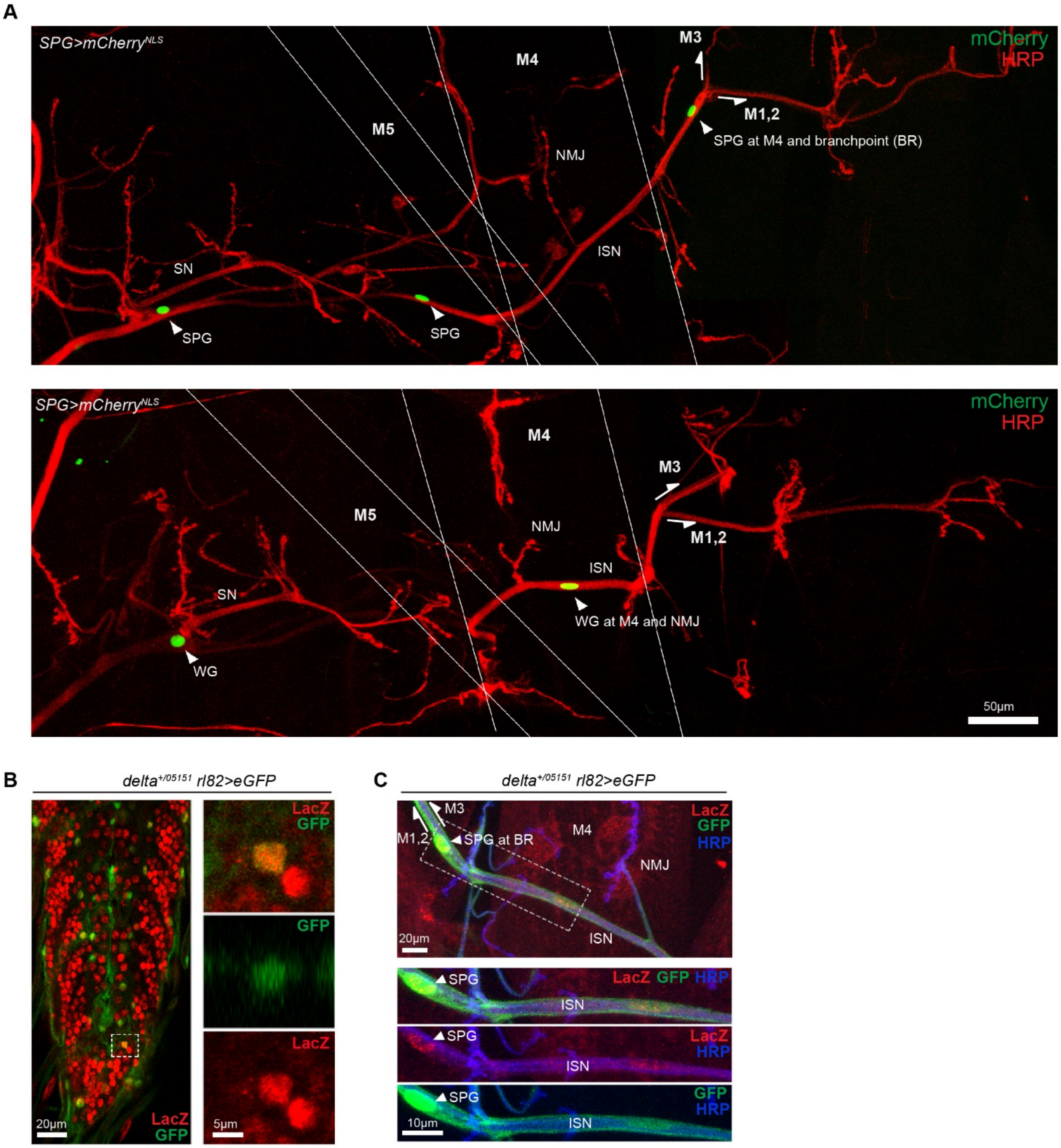
A
SPG>mCherry^NLS
mCherry
HRP
M3
M4
M5
M1,2
SPG at M4 and branchpoint (BR)
NMJ
ISN
SN
SPG
SPG
SPG>mCherry^NLS
mCherry
HRP
M4
M3
M5
NMJ
M1,2
ISN
SN
WG at M4 and NMJ
WG
50µm
B
delta+/05151 rl82>eGFP
LacZ
GFP
GFP
LacZ
20µm
LacZ
GFP
5µm
C
delta+/05151 rl82>eGFP
M3
M1,2
SPG at BR
M4
LacZ
GFP
HRP
NMJ
20µm
ISN
SPG
LacZ GFP HRP
ISN
SPG
LacZ
HRP
ISN
SPG
GFP
HRP
10µm
ISN

Figure S2. SPG nuclei are stereotypically located and express Delta. (A) Maximum intensity projection of a confocal Z-stack of the hemisegment of A3 showing SPG and WG nuclei. Top. Hemisegment of A3 of *UAS-mCherry*^{NLS};*moody-GAL4* stained with anti-HRP (red). Bottom. Hemisegment of A3 of *UAS-mCherry*^{NLS};*nrv2-GAL4* stained with anti-HRP (red). Arrowheads indicate the labeled nuclei. Muscle 4 and 5, and SN and ISN nerve bundles are labeled. (B) Single section of a confocal Z-stack of immunohistochemical staining showing the VNC of *rl82-GAL4;UAS-eGFP /delta*$^{+/05151}$ stained with anti-LacZ (red) along with a magnification of the selected area showing GFP and LacZ positive cells. (C) Maximum intensity projection of a confocal Z-stack of the nerve at the NMJ of A3M4 of *rl82-GAL4;UAS-eGFP/delta*$^{+/05151}$ stained with anti-LacZ (red) and anti-HRP (blue) along with a magnification of the selected area. The SPG nucleus in the vicinity of muscle 4 and the branch point is identified.

Figure S3

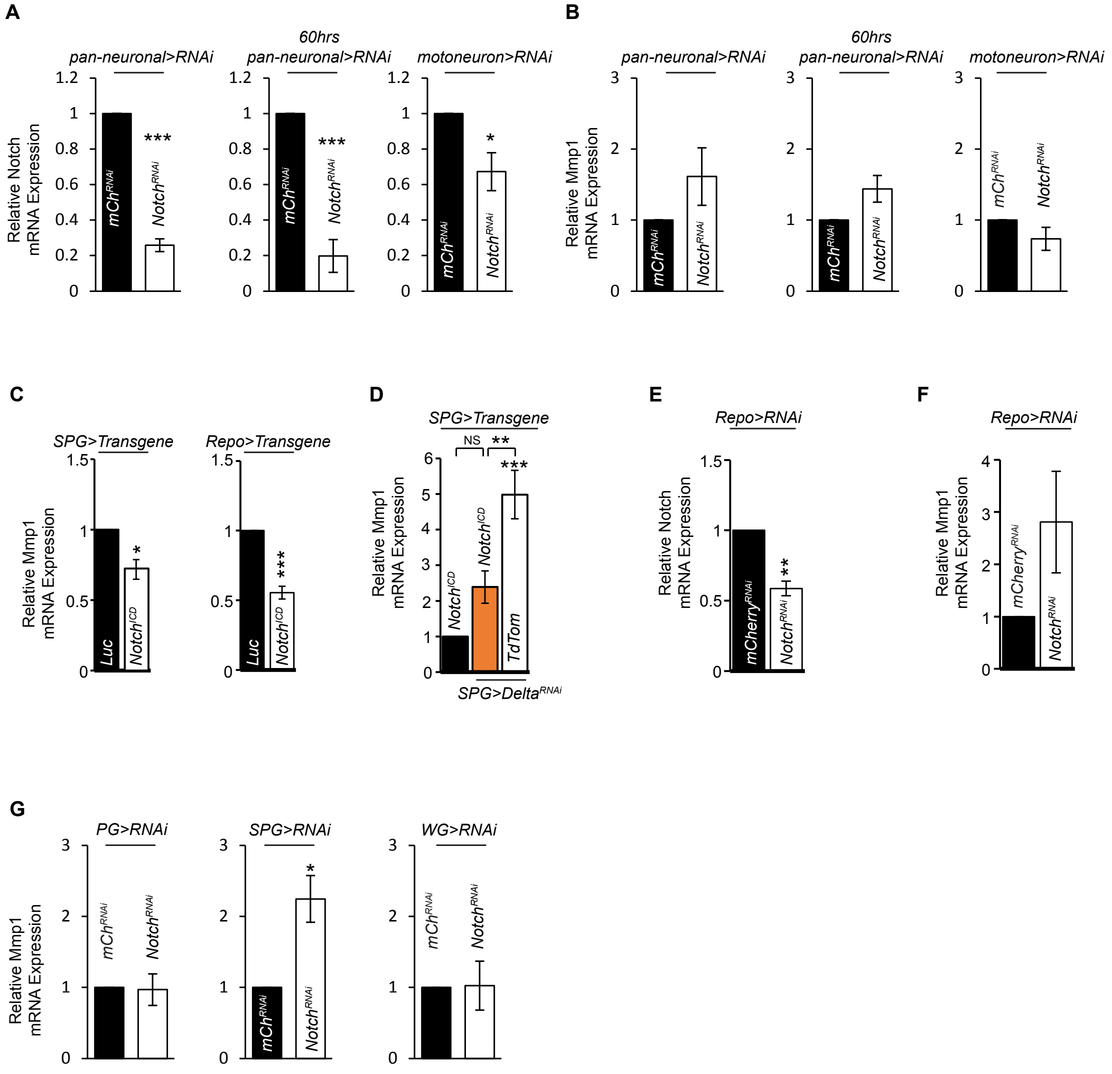
A
B
C
D
E
F
G
pan-neuronal>RNAi
60hrs
pan-neuronal>RNAi
motoneuron>RNAi
Relative Notch mRNA Expression
Relative Mmp1 mRNA Expression
mCh^RNAi
Notch^RNAi
***
*
SPG>Transgene
Repo>Transgene
Luc
Notch^ICD
NS
**
TdTom
SPG>Delta^RNAi
Repo>RNAi
mCherry^RNAi
PG>RNAi
SPG>RNAi
WG>RNAi

Figure S3. Notch regulates Mmp1 transcript levels. RT-qPCR analysis showing relative (A) *notch* or (B) *mmp1* mRNA expression in the CNS of *elav-GAL4;;UAS-mCherry*^{RNAi} or *UAS-Notch*^{RNAi}, *elav-GAL4; tub-GAL80*^{ts}*;UAS-mCherry*^{RNAi} or *UAS-Notch*^{RNAi} transferred to 29ºC for 60hrs, or *OK371;UAS-mCherry*^{RNAi} or *UAS-Notch*^{RNAi} , n=3 for each genotype followed by Student's T-test for each respective pair. (C) RT-qPCR analysis showing relative *mmp1* mRNA expression in the CNS of *moody-GAL4/UAS-Luciferase* or *UAS-Notch*^{ICD}, or *Repo-GAL4/UAS-Luciferase* or *UAS-Notch*^{ICD}, n=4 or n=3 respectively for each genotype followed by Student's T-test. (D) RT-qPCR analysis showing relative *mmp1* mRNA expression in the CNS of *UAS-Notch*^{ICD}*;moody-GAL4, UAS-Notch*^{ICD}*;moody-GAL4/UAS-Delta*^{RNAi}, or *UAS-TdTomato;moody-GAL4/UAS-Delta*^{RNAi}, n=5 for each genotype followed by a one-way ANOVA and Tukey's test for multiple comparisons. RT-qPCR analysis showing relative (E) *notch* or (F) *mmp1* mRNA expression in the CNS of *Repo-GAL4/UAS-mCherry*^{RNAi} or *UAS-Notch*^{RNAi}, n=3 for each genotype followed by Student's T-test. (G) RT-qPCR analysis showing relative *mmp1* mRNA expression in the CNS of (PG) *c527-GAL4* (n=3), (SPG) *moody-GAL4* (n=3), (WG) *nrv2-GAL4* (n=3) crossed to *UAS-mCherry*^{RNAi} or *UAS-Notch*^{RNAi} followed by Student's T-test for each respective pair.

Figure S4

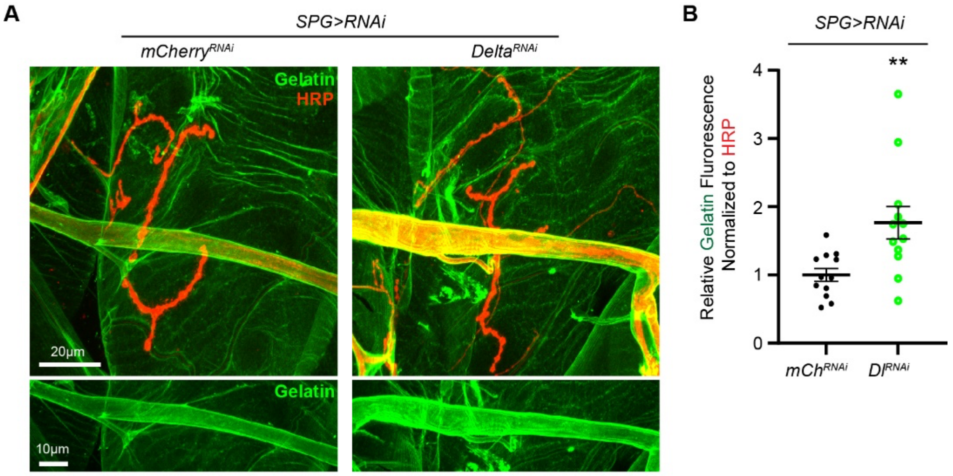

Figure S4. Increased proteolytic activity on the nerve following Delta knockdown in SPG. (A) Maximum intensity projection of a confocal Z-stack of the nerve region of A3M4 of *Moody-GAL4/UAS-mCherry*^{RNAi} or *UAS-Delta*^{RNAi} incubated with Gelatin (green) and stained with anti-Hrp (red). (B) Quantification of Gelatin fluorescence signal intensity in (A), n=12 for each genotype followed by Student's T-test.

Figure S5

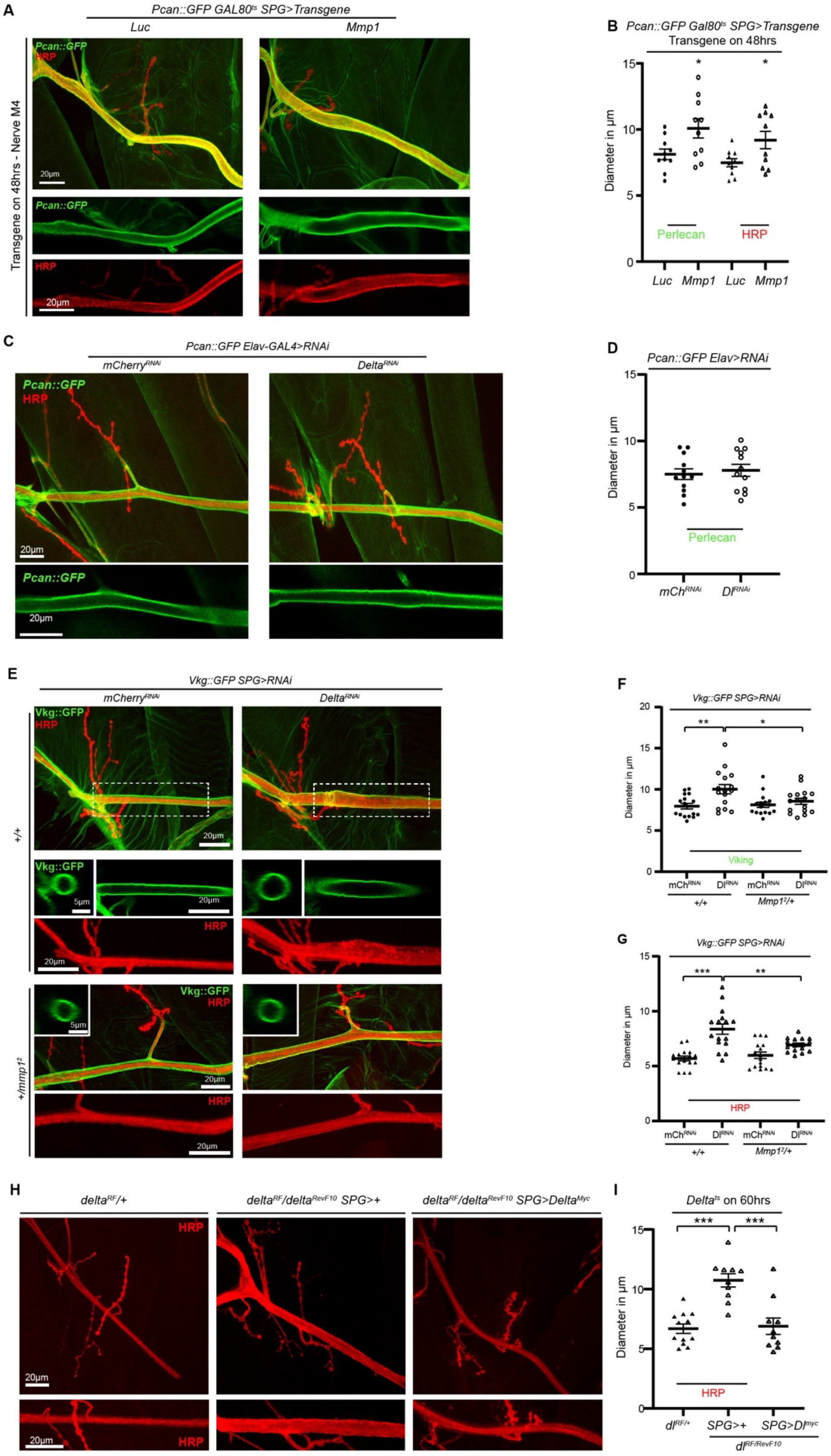
A
Pcan::GFP GAL80ts SPG>Transgene
Luc
Mmp1
Transgene on 48hrs - Nerve M4
Pcan::GFP
HRP
20µm
B Pcan::GFP Gal80ts SPG>Transgene
Transgene on 48hrs
Diameter in µm
Perlecan
HRP
Luc
Mmp1
C
Pcan::GFP Elav-GAL4>RNAi
mCherryRNAi
DeltaRNAi
D Pcan::GFP Elav>RNAi
mChRNAi
DlRNAi
E
Vkg::GFP SPG>RNAi
Vkg::GFP
5µm
+/+
+/mmp12
F
G
Viking
Mmp12/+
H
deltaRF/+
deltaRF/deltaRevF10 SPG>+
deltaRF/deltaRevF10 SPG>DeltaMyc
I
Deltats on 60hrs
dlRF/+
SPG>+
SPG>Dlmyc
dlRF/RevF10

Figure S5. Delta-deficient SPG cause nerve expansion through Mmp1. (A) Top. Maximum intensity projection of a confocal Z-stack of the larval motor nerve at the NMJ of A3M4 of *Pcan::GFP;GAL80^{ts};UAS-Luciferase* or *UAS-Mmp1* transferred to 27º C for 48hrs and stained with anti-Hrp (red). Bottom. Orthogonal view of and single section of the confocal Z-stack showing only the Pcan::GFP or HRP signal. (B) Diameter of the nerve as delineated by Pcan::GFP, or HRP for the genotypes in (A), n=10 for each genotype followed by Student's T-test for each respective pair. (C) Maximum intensity projection of a confocal Z-stack of the larval motor nerve at the NMJ of A3M4 of *Elav-GAL4/Pcan::GFP;;UAS-mCherry^{RNAi}* or *UAS-Delta^{RNAi}* stained with anti-Hrp (red). Bottom. Orthogonal view of and single section of the confocal Z-stack showing only the Pcan::GFP signal. (D) Diameter of the nerve as delineated by Pcan::GFP for the genotypes in (C), n=14 for each genotype followed by Student's T-test.

(E) Top. Maximum intensity projection of a confocal Z-stack of the larval nerve at the NMJ of A3M4 of *Vkg::GFP;moody-GAL4/UAS-mCherry^{RNAi}* or *UAS-Delta^{RNAi}* stained with anti-HRP (red) with orthogonal views of a single section of the confocal Z-stack from the selected area showing only the Vkg::GFP signal, and the maximum intensity projection of the confocal Z-stack of the HRP signal. Bottom. Maximum intensity projection of a confocal Z-stack of the larval nerve at the NMJ of A3M4 of *mmp1^{2}/Vkg::GFP;moody-GAL4/UAS-mCherry^{RNAi}* or *UAS-Delta^{RNAi}* stained with anti-HRP (red). Inset shows orthogonal view of the Vkg::GFP signal of the confocal Z-stack of the nerve, with the maximum intensity projection of the confocal Z-stack of the HRP signal. (F) Diameter of the nerve as delineated by Vkg::GFP for the genotypes in (E) n=14 for *wild type* and n=16 for *mmp1 het* followed by a one-way ANOVA and Tukey's test for multiple comparisons. (G) Diameter of the nerve as delineated by HRP for the corresponding genotypes in (E) followed by a one-way ANOVA and Tukey's test for multiple comparisons.

(H) Top. Maximum intensity projection of a confocal Z-stack of the larval motor nerve at the NMJ of A3M4 of *delta$^{RF/+}$*, *Moody-GAL4/+;delta$^{RF/RevF10}$*, and *Moody-GAL4/UAS-Delta^{myc};delta$^{RF/RevF10}$* transferred to 29º C for 60hrs and stained with anti-Hrp (Red). Bottom. Magnification of the HRP signal. (I) Diameter of the nerve as delineated by HRP for the genotypes in (H), n=12 for *delta het*, n=10 for *delta mutant* and *delta mutant* with delta expressed in SPG followed by a one-way ANOVA and Tukey's test for multiple comparisons.

Figure S6

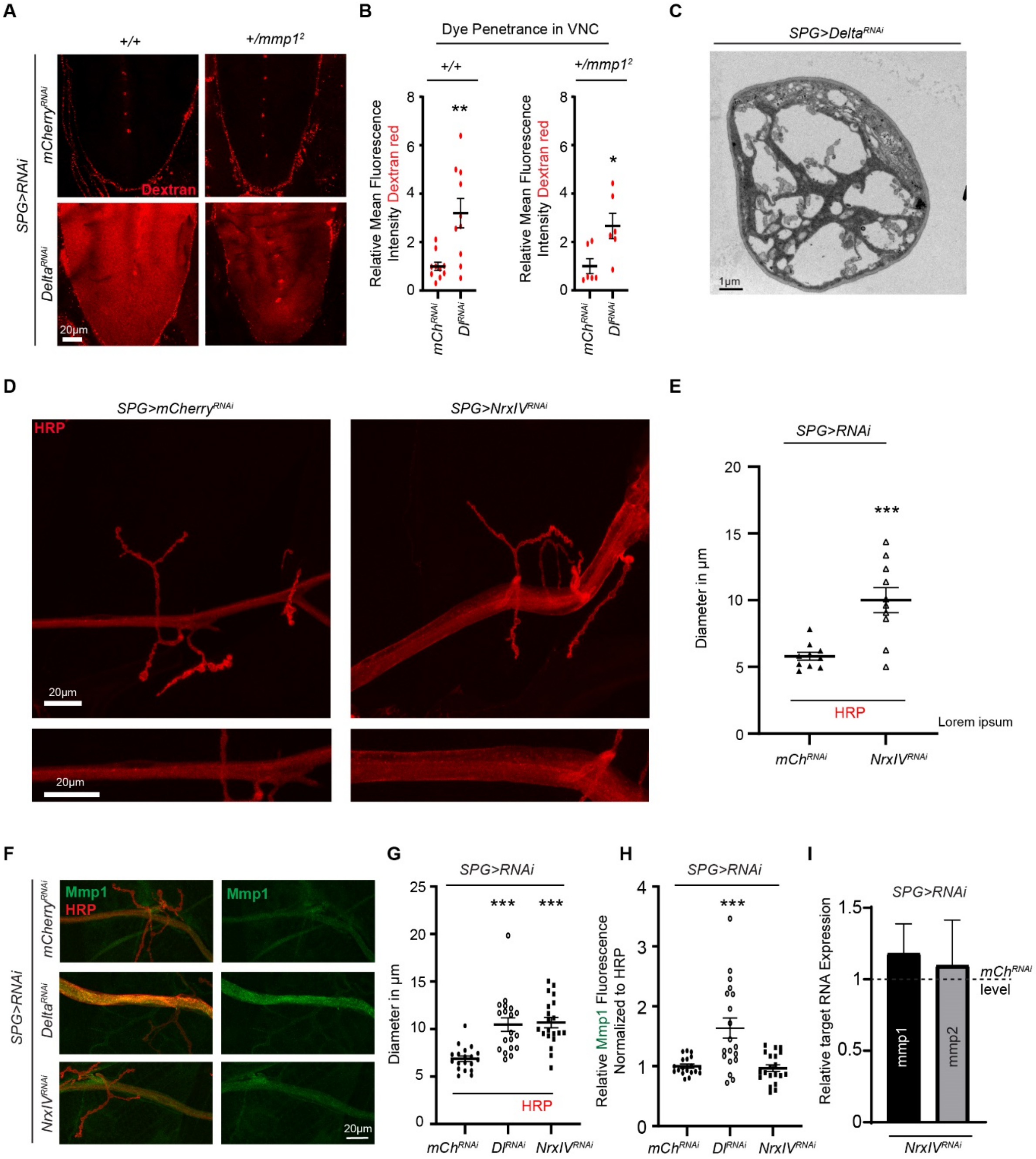

A
+/+
+/mmp1²
SPG>RNAi
mCherryRNAi
DeltaRNAi
Dextran
20µm
B
Dye Penetrance in VNC
+/+
+/mmp1²
Relative Mean Fluorescence Intensity Dextran red
mChRNAi
DlRNAi
C
SPG>DeltaRNAi
1µm
D
SPG>mCherryRNAi
SPG>NrxIVRNAi
HRP
20µm
E
SPG>RNAi
Diameter in µm
HRP
Lorem ipsum
mChRNAi
NrxIVRNAi
F
Mmp1
HRP
SPG>RNAi
mCherryRNAi
DeltaRNAi
NrxIVRNAi
20µm
G
SPG>RNAi
Diameter in µm
HRP
mChRNAi
DlRNAi
NrxIVRNAi
H
SPG>RNAi
Relative Mmp1 Fluorescence Normalized to HRP
mChRNAi
DlRNAi
NrxIVRNAi
I
SPG>RNAi
Relative target RNA Expression
mmp1
mmp2
mChRNAi level
NrxIVRNAi

Figure S6. Delta knockdown in SPG disrupts barrier function. (A) Single section of a confocal Z-stack showing the VNC of *moody-GAL4/UAS-mCherry*^{RNAi} or *UAS-Delta*^{RNAi} (left) or *mmp1*$^{+/2}$;*moody-GAL4/UAS-mCherry*^{RNAi} or *UAS-Delta*^{RNAi} (right) following injection with Dextran (red). (B) Quantification of the Dextran fluorescence intensity in (A) n=10 for each *wt* genotype, and n=6 for each *mmp1 het* genotype followed by Student's T-test for each respective pair. (C) Ultrastructure of third instar larval nerve of *moody-GAL4/UAS-Delta*$^{RNAi.}$.The nerve diameter is larger in Delta knockdown, and large vacuole-like structures can be detected that have broken down the nerve. (D) Top. Maximum intensity projection of a confocal Z-stack of the larval motor nerve at the NMJ of A3M4 of *moody-GAL4/UAS-mCherry*^{RNAi} or *UAS-NrxIV*^{RNAi} stained with anti-Hrp (red). Bottom. Magnification of the HRP signal. (E) Diameter of the nerve as delineated by HRP for the genotypes in (D), n=10 for each genotype followed by Student's T-test. (F) Maximum intensity projection of a confocal Z-stack of the larval motor nerve at the NMJ of A3M4 of *mody-GAL4/UAS-mCherry*^{RNAi}*, Delta*^{RNAi}*, or NrxIV*^{RNAi} stained with anti-Hrp (red) and anti-Mmp1 (green). (G) Diameter of the nerve as delineated by HRP for the genotypes in (F), n=19 for *mCherry*^{RNAi}, n=19 for *Dl*^{RNAi}, and n=20 for *Nrx*^{RNAi} followed by a one-way ANOVA and Tukey's test for multiple comparisons. (H) Quantification of the anti-Mmp1 fluorescence signal intensity of the nerve in (G), n=19 for *mCherry*^{RNAi} n=19 for *Dl*^{RNAi}, and n=20 for *Nrx*^{RNAi} followed by a one-way ANOVA and Tukey's test for multiple comparisons. (I) RT-qPCR analysis showing *mmp1* and *mmp2* expression in the CNS of *moody-GAL4;UAS-mCherry*^{RNAi} or *UAS-NrxIV*^{RNAi}*,* n=5 for each genotype followed by Student's T-test for each respective target. Note mCherry^{RNAi} is not depicted.

Figure S7

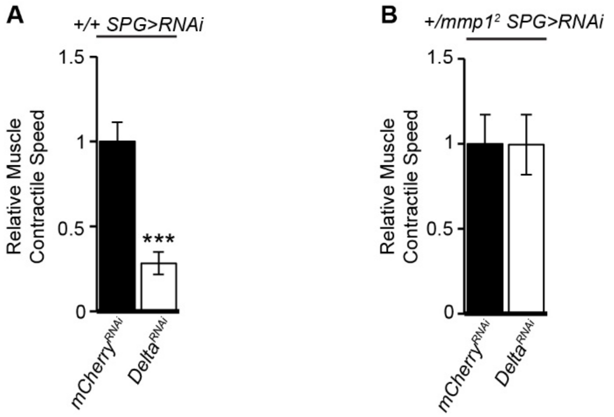

Figure S7. Delta knockdown disrupts coordinated muscle contractions. (A) Quantification of muscle contractions recorded in time-lapse images of *moody-GAL4/UAS-mCherry*^{RNAi} (n=8) or *UAS-Delta*^{RNAi} (n=10) after dissection in HL-3 and incubated in high K^+ HL-3, adjusted to maintain osmolarity (in mM) followed by Student's T-test. See materials and methods for complete details. (B) Quantification of muscle contractions recorded in time-lapse images of *mmp1*$^{+/2}$;*moody-GAL4/UAS-mCherry*^{RNAi} (n=10) or *UAS-Delta*^{RNAi} (n=10) after dissection in HL-3 and incubated in high K^+ HL-3, adjusted to maintain osmolarity (in mM) followed by Student's T-test.

Figure S8

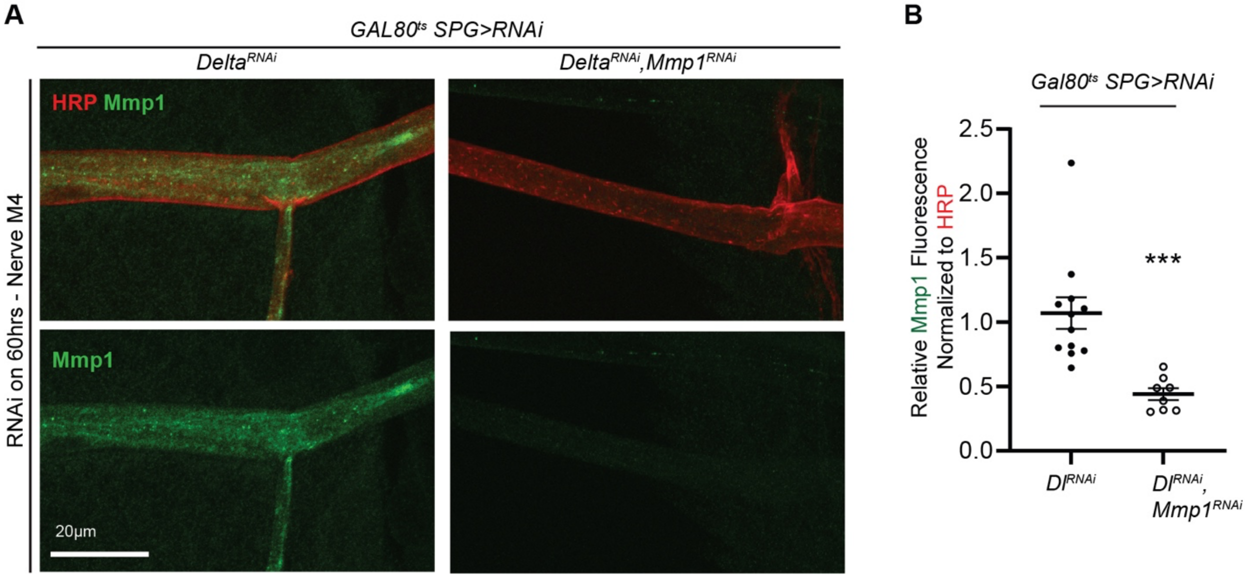

Figure S8. Knockdown of *mmp1* in SPG rescues barrier function and nerve structure in *Delta* knockdown. (A) Maximum intensity projection of a confocal Z-stack at the larval motor nerve at the NMJ of A3M4 of *tub-GAL80^{ts}/moody-GAL4;UAS-Delta^{RNAi}* or *tubGAL80^{ts}/moody-GAL4;UAS-Delta^{RNAi}/UAS-Mmp1^{RNAi}* stained with anti-HRP (red) and anti-Mmp1 (green). (B) Quantification of relative anti-Mmp1 fluorescence signal intensities of the nerve at A3M4 for genotypes in (A), n=12 for Delta^{RNAi}, n=8 for Delta^{RNAi}, Mmp1^{RNAi} followed by Student's T-test.

Figure S9

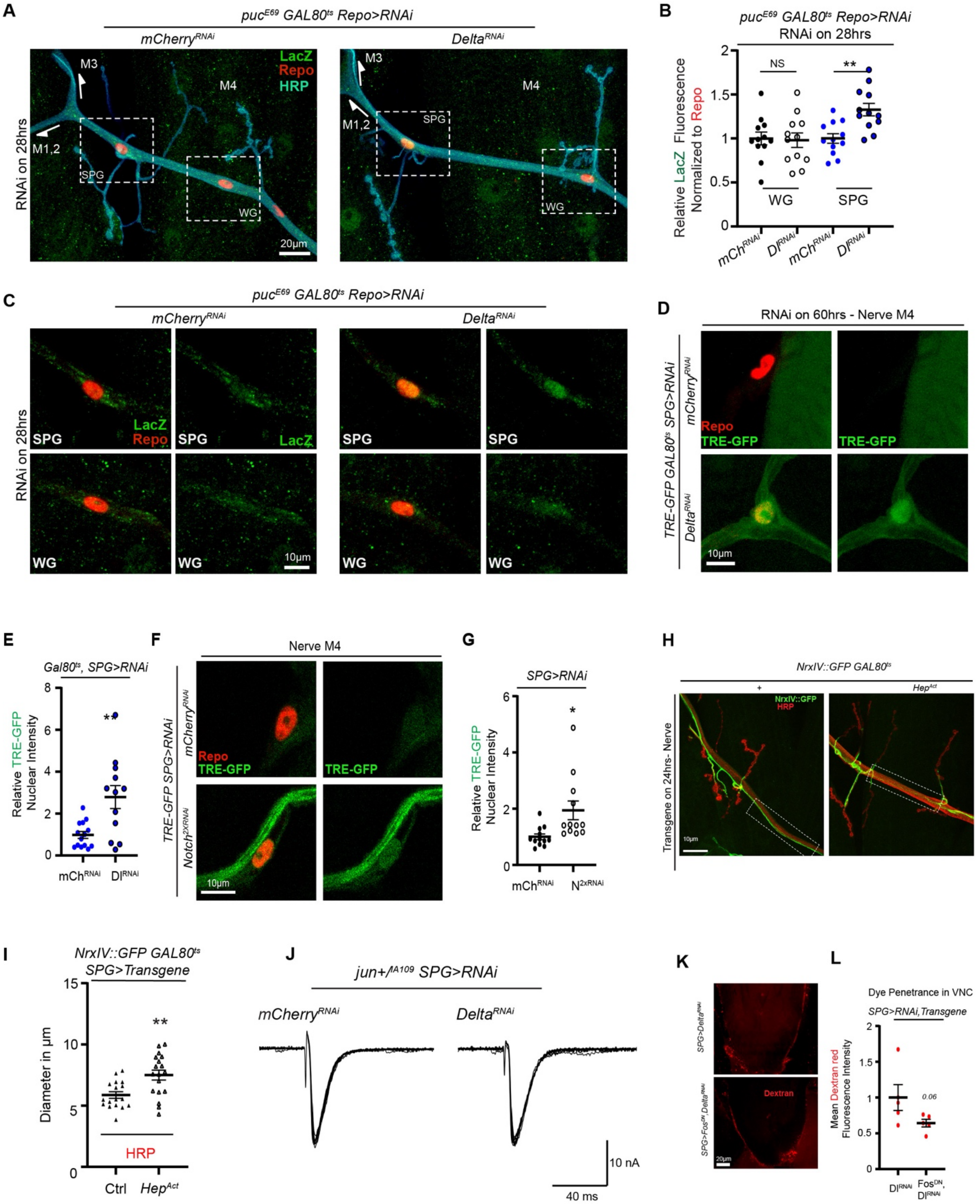
A
puc^E69 GAL80^ts Repo>RNAi
mCherry^RNAi
Delta^RNAi
LacZ
Repo
HRP
M3
M4
M1,2
SPG
WG
RNAi on 28hrs
20µm
B
puc^E69 GAL80^ts Repo>RNAi
RNAi on 28hrs
NS
**
Relative LacZ Fluorescence Normalized to Repo
WG
SPG
mCh^RNAi
Dl^RNAi
C
puc^E69 GAL80^ts Repo>RNAi
mCherry^RNAi
Delta^RNAi
RNAi on 28hrs
LacZ
Repo
SPG
WG
10µm
D
RNAi on 60hrs - Nerve M4
TRE-GFP GAL80^ts SPG>RNAi
mCherry^RNAi
Delta^RNAi
Repo
TRE-GFP
10µm
E
Gal80^ts, SPG>RNAi
Relative TRE-GFP Nuclear Intensity
**
mCh^RNAi
Dl^RNAi
F
Nerve M4
TRE-GFP SPG>RNAi
mCherry^RNAi
Notch^2XRNAi
Repo
TRE-GFP
10µm
G
SPG>RNAi
Relative TRE-GFP Nuclear Intensity
*
mCh^RNAi
N^2xRNAi
H
NrxIV::GFP GAL80^ts
+
Hep^Act
NrxIV::GFP
HRP
Transgene on 24hrs- Nerve
10µm
I
NrxIV::GFP GAL80^ts
SPG>Transgene
Diameter in µm
**
HRP
Ctrl
Hep^Act
J
jun+/^IA109 SPG>RNAi
mCherry^RNAi
Delta^RNAi
10 nA
40 ms
K
SPG>Delta^RNAi
SPG>Fos^DN Delta^RNAi
Dextran
20µm
L
Dye Penetrance in VNC
SPG>RNAi,Transgene
Mean Dextran red Fluorescence Intensity
0.06
Dl^RNAi
Fos^DN, Dl^RNAi

Figure S9. Negative regulation of Mmp1 by Delta/Notch in SPG is JNK dependent.

(A) Maximum intensity projection of a confocal Z-stack of the motor nerve at the NMJ of A3M4 of *tub-GAL80^{ts};Repo-GAL4,puc^{E69}/UAS-mCherry^{RNAi}* or *UAS-Delta^{RNAi}* transferred to 29ºC for 28hrs and stained with anti-LacZ (green), anti-Repo (red), and anti-HRP (cyan). Nuclei of subperineurial glia (SPG) and wrapping glia (WG) are marked. (B) Quantification of the LacZ relative fluorescence intensity in (B), n=12 for mCherry^{RNAi} and Delta^{RNAi} followed by Student's T-test for each respective pair. (C) Maximum intensity projections of the magnification of the selected areas in A.

(D) Maximum intensity projection of a confocal Z-stack of the SPG nucleus on the motor nerve bundle of the branch point at M4 of *TRE-GFP/moody-GAL4;tub-GAL80^{ts}/UAS-mCherry^{RNAi}* or *UAS-Delta^{RNAi}* transferred to 29ºC for 60hrs and stained with anti-Repo (red). (E) Quantification of the GFP fluorescence intensity normalized to Repo in (A), n=14 nuclei for mCherry^{RNAi} and n=12 nuclei for Delta^{RNAi} followed by Student's T-test. (F) Maximum intensity projection of a confocal Z-stack of the SPG nucleus on the motor nerve bundle of the branch point at M4 of *TRE-GFP;moody-GAL4/UAS-mCherry^{RNAi}* or *UAS-Notch^{RNAi};TRE-GFP;moody-GAL4/ UAS-Notch^{RNAi}* stained with anti-Repo (red). (G) Quantification of the GFP fluorescence intensity normalized to Repo in (F) n=12 nuclei for each genotype followed by Student's T-test.

(H) Maximum intensity projection of a confocal Z-stack of the nerve at NMJ A3M4 of *tub-GAL80^{ts};NrxIV::GFP/moody-GAL4* or *tub-GAL80^{ts}/UAS-Hep^{Act};NrxIV::GFP/moody-GAL4* transferred to 29ºC for 28hrs and stained with anti-HRP. Selection is shown in main Figure 6. (I) Diameter of the nerve as delineated by HRP for the genotypes in (H), n=16 for control and n=18 for Hep^{Act} followed by Student's T-test.

(J) Representative traces of EPSCs from *jun$^{+/IA109}$;moody-GAL4/UAS-mCherry^{RNAi} or UAS-Delta^{RNAi}*.

(K) Single section of a confocal Z-stack showing the VNC of *moody-GAL4/UAS-Delta^{RNAi}* or *UAS-Fos^{DN};moody-GAL4/UAS-Delta^{RNAi}* following injection with Dextran (red). (L) Quantification of the Dextran mean fluorescence intensity signal in (K) n=5 for each genotype followed by Student's T-test.

Table S1. Ligand Screen
mRNA Relative Fold Change to mCherry^{RNAi}

	Mmp1		Mmp2	
	**Mean±SEM**	**P-value**	**Mean±SEM**	**P-value**
**BMP signaling pathway**				
Gbb^{RNAi}	1.368±0.61	1.00	1.063±0.27	1.00
Mav^{RNAi}	2.151±0.35	0.95	1.652±0.27	0.72
**Notch signaling pathway**				
Dl^{RNAi}	12.27±2.11	$4.7x10^{-4}$	0.471±0.06	0.91
Ser^{RNAi}	0.853±0.04	1.00	0.913±0.18	1.00
**Neurotrophins**				
Nt1^{RNAi}	1.301±0.25	1.00	0.902±0.06	1.00
Spz^{RNAi}	1.238±0.07	1.00	0.798±0.15	0.99
Manf^{RNAi}	1.610±0.47	1.00	0.939±0.24	1.00
**Cell-Cell/Matrix Interactions**				
Tsp^{RNAi}	1.433±0.24	1.00	0.942±0.16	1.00
**TGFα-like signaling pathway**				
Grk^{RNAi}	0.953±0.07	1.00	0.651±0.19	1.00
Spi^{RNAi}	1.603±0.72	1.00	1.124±0.26	1.00
**TGFβ-like signaling pathway**				
Actβ^{RNAi}	1.924±0.87	0.99	0.936±0.21	1.00
Daw^{RNAi}	0.773±0.32	1.00	0.628±0.33	1.00
Myo^{RNAi}	2.402±0.82	0.82	1.091±0.25	1.00
**Neuregulin-like signaling pathway**				
Vn^{RNAi}	0.879±0.18	1.00	1.157±0.17	1.00
**Wnt/Wg signaling pathway**				
Wg^{RNAi}	1.295±0.46	1.00	1.218±0.29	1.00
**Cytokine Signaling pathway**				
Upd1^{RNAi}	0.656±0.1	1.00	0.890±0.27	1.00
Upd2^{RNAi}	3.008±1.4	0.36	1.911±0.81	0.28
Upd3^{RNAi}	1.646±0.69	1.00	1.432±0.14	0.98
**FGF-like signaling pathway**				
Bnl^{RNAi}	1.394±0.58	1.00	1.439±0.46	0.98
Pyr^{RNAi}	2.302±0.47	0.88	1.421±0.31	0.99
**Sex Determination pathway**				
To^{RNAi}	0.472±0.22	1.00	0.618±0.38	0.99
**Immunoglobulin-like signaling pathway**				
Tutl^{RNAi}	0.385±0.16	1.00	0.454±0.22	0.89
**LDL-like signaling pathway**				
Jeb^{RNAi}	1.166±0.38	1.00	0.740±0.08	1.00
**Thioredoxin-like signaling pathway**				
Prtp^{RNAi}	1.073±0.42	1.00	1.133±0.1	1.00

Screen for potential glial ligands that alter mRNA expression of Mmp1 or Mmp2. Relative Mmp1 or Mmp2 mRNA expression in third instar larval CNS of *repo-GAL4/UAS-mCherry*^{RNAi} or *UAS-Ligand*^{RNAi}. n=3 for each genotype followed by a one-way ANOVA with Tukey's test for multiple comparisons. Bone morphogenic protein (*glass bottom boat*, *maverick*), Notch signaling (*delta*, *serrate*), Neurotrophin pathways (*neurotrophin 1*, *spatzle*, *mesencephalic astrocyte-derived neurotrophic factor*), cell-cell matrix interaction (*thrombospondin*), Transforming growth factor (*activinβ*, *dawdle*, *myoglianin*), Neuregulin-like (*vein*), Wingless signaling (*wg*), Jak/STAT signaling (*unpaired 1, 2, 3*), FGF-like pathway (*branchless, pyramus*), Sex determination (*takeout*), Immunoglobulin-like (*turtle*), LDL-like (*jelly belly*), and Thioredoxin-like (*pretaporter*).

Table S2. List of GAL4 drivers used

**Tissue expression**	**Driver**
Pan-neuronal	*Elav-GAL4*
Motoneurons	*OK371-GAL4 (vGLUT),*<br>*BG380-GAL4*
Pan-glial	*Repo-GAL4* (X & 3rd chr)
Cortex glial	*GMR54H02-GAL4*
Astrocyte-like glia	*Alrm-GAL4*
Ensheathing glial	*GMR56F03-GAL4*
Peineurial glia	*c527-GAL4*
Subperineurial glia	*moody-GAL4* (2nd & 3rd chr)<br>*rl82-GAL4 (Gliotactin)*
Wrapping glia	*nrv2-GAL4*

Table S3 Electrophysiology Data

Mean & s.e.m are shown. Student's T-test was applied for all pair wise comparisons

Data for Figure 4G

Genotype	mEPSC (nA)	EPSC (nA)	QC	n
moody-GAL4/UAS-mCherry^{RNAi}	0.89±0.03	22.95±2.39	26.53±3.11	13
moody-GAL4/UAS-Delta^{RNAi}	0.92±0.02	12.23±2.02	13.21±2.26	14
	P=0.39	*P=0.002*	*P=0.002*	

Data for Figure 4H

Genotype	mEPSC (nA)	EPSC (nA)	QC	n
delta^{RF}/+	0.55±0.02	21.98±2.43	39.83±3.94	12
delta^{RF}/delta^{RevF10}	0.52±0.03	13.94±2.65	27.32±5.57	10
	P=0.46	*P=0.04*	*P=0.08*	

Data for Figure 4I

Genotype	mEPSC (nA)	EPSC (nA)	QC	n
alrm-GAL4;UAS-mCherry^{RNAi}	0.76±0.04	20.47±1.74	27.29±2.22	14
alrm-GAL4;UAS-Delta^{RNAi}	0.80±0.04	22.95±2.6	29.79±4.16	12
	P=0.51	*P=0.60*	*P=0.64*	

Data for Figure 4K

Genotype	mEPSC (nA)	EPSC (nA)	QC	n
mmp1$^{+/2}$;moody-GAL4/UAS-mCherry^{RNAi}	0.82±0.06	22.24±3.6	27.85±4.49	9
mmp1$^{+/2}$;moody-GAL4/UAS-Delta^{RNAi}	0.78±0.03	18.69±3.03	24.39±4.4	7
	P=0.57	*P=0.48*	*P=0.60*	

Data for Figure 5E

Genotype	mEPSC (nA)	EPSC (nA)	QC	n
moody-GAL4/+	0.80±0.02	28.35±2.60	35.76±3.59	15
moody-GAL4/+;UAS-Mmp1^{RNAi}/+	0.77±0.02	28.51±2.38	37.87±3.81	13
	P=0.33	*P=0.97*	*P=0.69*	

Data for Figure 5F

Genotype	mEPSC (nA)	EPSC (nA)	QC	n
moody-GAL4/tubGal80^{ts};UAS-Delta^{RNAi}/+	0.72±0.01	11.52±2.40	16.03±3.30	15
moody-GAL4/tubGal80^{ts};UAS-Delta^{RNAi}/UAS-Mmp1^{RNAi}	0.72±0.01	26.07±2.42	36.5±3.619	17
	P=0.758	*P<0.001*	*P<0.001*	

Data for Figure 6H

Genotype	mEPSC (nA)	EPSC (nA)	QC	n
jun$^{+/IA109}$;moody-GAL4/UAS-mCherry^{RNAi}	0.75±0.02	16.94±2.66	22.46±3.31	12
jun$^{+/A109}$;moody-GAL4/UAS-Delta^{RNAi}	0.73±0.03	18.54±2.87	25.76±4.12	13
	P=0.60	*P=0.69*	*P=0.54*	

Table S4 Fly Stocks

REAGENT or RESOURCE	SOURCE	IDENTIFIER
D. Melanogaster. rl82-GAL4: P{GawB}Gli^{rL82}	Gift from V.Budnik. Sepp et Auld. 1999	FBal0181665
D. Melanogaster. UAS-eGFP	Maksoud et al. 2019	
D. Melanogaster. repo-GAL4: w[1118]; P{w[+m*]=GAL4}repo	Bloomington Drosophila Stock Center	BDSC:7415; FlyBase: FBst0007415
D. Melanogaster. RNAi for mCherry: y[1] sc[*] v[1] sev[21]; P{y[+t7.7] v[+t1.8]=VALIUM20-mCherry}attP2	Bloomington Drosophila Stock Center	BDSC:35785; FlyBase: FBst0035785
D. Melanogaster. RNAi for Delta: y[1] sc[*] v[1] sev[21]; P{y[+t7.7] v[+t1.8]=TRiP.HMS01309}attP2	Bloomington Drosophila Stock Center	BDSC:34322; FlyBase: FBst0034322
D. Melanogaster. elav-GAL4: P{GawB}elav^{C155}	Bloomington Drosophila Stock Center	BDSC:458; FlyBase: FBal0047071
D. Melanogaster. ok371-GAL4: w[1118]; P{w[+mW.hs]=GawB}VGlut[OK371]	Bloomington Drosophila Stock Center	BDSC:26160; FlyBase: FBst0026160
D. Melanogaster. c527-GAL4: P{GawB}c527	Gift from R. Ordway (Hummel et al. 2002)	
D. Melanogaster. Moody-GAL4 (3rd chr.): P{moody-GAL4}	Gift from R.Ordway (Schwabe et al. 2005)	
D. Melanogaster. Moody-GAL4 (2nd chr.): P{moody-GAL4.SPG}	Gift from V. Auld (Schwabe et al. 2005)	
D. Melanogaster. Nrv2-GAL4: w*; P{nrv2-GAL4.S}8	Bloomington Drosophila Stock Center	BDSC: 6799 Flybase FBst0006799
D. Melanogaster. tub-GAL80^{ts} (2nd chr.). w[*]; P{w[+mC]=tubP-GAL80[ts]}20	Bloomington Drosophila Stock Center	BDSC: 7019 Flybase FBst0007019
D. Melanogaster. Repo-GAL4 (X chr.)	Karla Kaun	
D. Melanogaster. UAS-Dcr2: w[1118]; P{w[+mC]=UAS-Dcr-2.D}2	Bloomington Drosophila Stock Center	BDSC: 24650 Flybase FBst0024650
D. Melanogaster. RNAi for Delta. w[1118]; P{GD2642}v37287	Vienna Drosophila Resource Center	VDRC: 37287 Flybase FBst0461934
D. Melanogaster. Delta^{RF}: Dl[RF]	Bloomington Drosophila Stock Center	BDSC: 5603 Flybase FBst0005603
D. Melanogaster. Delta^{RevF10}: Dl[RevF10] e[*] P{ry[+t7.2]=neoFRT}82B	Bloomington Drosophila Stock Center	BDSC: 6300 Flybase FBst0006300
D. Melanogaster. Perlecan::GFP	Gift from V. Auld (Xie & Auld 2011)	
D. Melanogaster. NrxIV::GFP: y[1] w[*]; P{w[+mC]=PTT-GA}Nrx-IV[CA06597]	Bloomington Drosophila Stock Center	BDSC: 50798 Flybase FBst0050798
D. Melanogaster. Viking::GFP	Gift from T. Kornberg. (Buszczak et al. 2007)	

D. Melanogaster. UAS-mCD8::RFP	Gift from V. Auld (Xie & Auld 2011)	
D. Melanogaster. RNAi for Luciferase. y[1] v[1]; P{y[+t7.7] v[+t1.8]=TRiP.JF01355}attP2	Bloomington Drosophila Stock Center	BDSC: 31603 Flybase FBst0031603
D. Melanogaster. Alrm-GAL4 (2nd chr)	M. Freeman (Stork et al. 2014)	
D. Melanogaster. $Mmp1^{2}$: w[*]; Mmp1[2]	Bloomington Drosophila Stock Center	BDSC: 58709 Flybase FBst0058709
D. Melanogaster. RNAi for NrxIV. y[1] sc[*] v[1] sev[21]; P{y[+t7.7] v[+t1.8]=TRiP.HMS00419}attP2	Bloomington Drosophila Stock Center	BDSC: 32424 Flybase FBst0032424
D. Melanogaster. UAS-$Notch^{ICD}$. (2nd & 3rd chr.)	Gift from T. Nystul (Johnston et al. 2016)	
D. Melanogaster. RNAi for Notch. y[1] v[1]; P{y[+t7.7] v[+t1.8]=TRiP.HMS00009}attP2	Bloomington Drosophila Stock Center	BDSC:33616; FlyBase: FBst0033616
D. Melanogaster. RNAi for Notch. P{w[+mC]=UAS-N.dsRNA.P}14E, w[*]	Bloomington Drosophila Stock Center	BDSC:7078; FlyBase: FBst0007078
D. Melanogaster. UAS-TdTom. w[*]; P{w[+mC]=UAS-tdTom.S}2	Bloomington Drosophila Stock Center	BDSC:36327; FlyBase: FBst0036327
D. Melanogaster. Puc^{E69}. Puc[E69]	Gift from H. Jasper. (Wang et al. 2005)	
D. Melanogaster. Jun^{IA109}. cn[1] Jra[IA109] bw[1] sp[1]	Bloomington Drosophila Stock Center	BDSC:3273; FlyBase: FBst0003273
D. Melanogaster. Bsk^{1}. bsk[1] cn[1] bw[1] sp[1]	Bloomington Drosophila Stock Center	BDSC:3088; FlyBase: FBst0003088
D. Melanogaster. UAS-Fos^{DN}. w[1118]; P{w[+mC]=UAS-Fra.Fbz}5	Bloomington Drosophila Stock Center	BDSC:7214; FlyBase: FBst0007214
D. Melanogaster. UAS-Hep^{act}.	Bloomington Drosophila Stock Center	BDSC:9306; FlyBase: FBst0009306
D. Melanogaster. TRE::GFP. w[*]; P{y[+t7.7] w[+mC]=TRE-EGFP}attP16	Bloomington Drosophila Stock Center	BDSC:59010; FlyBase: FBst0059010
D. Melanogaster. tub-$GAL80^{ts}$ (3rd chr.).	Bloomington Drosophila Stock Center	BDSC:7017; FlyBase: FBst0007017
D. Melanogaster. UAS-Luciferase. y[1] v[1]; P{y[+t7.7] v[+t1.8]=UAS-LUC.VALIUM10}attP2	Bloomington Drosophila Stock Center	BDSC:35788; Flybase: FBst0035788
D. Melanogaster. UAS-Mmp1. w[*]; P{w[+mC]=UAS-Mmp1.f1}3	Bloomington Drosophila Stock Center	BDSC:58701; Flybase: FBst0058701
D. Melanogaster. GMR56F03-GAL4. w[1118]; P{y[+t7.7] w[+mC]=GMR56F03-GAL4}attP2	Bloomington Drosophila Stock Center	BDSC:39157; Flybase: FBst0039157

D. Melanogaster. GMR54H02-GAL4. w[1118]; P{y[+t7.7] w[+mC]= GMR54H02-GAL4}attP2	Bloomington Drosophila Stock Center	BDSC:45784; Flybase: FBst0045784
D. Melanogaster BG380-GAL4.	Gift from C. Goodman. (Budnik et al. 1996)	
D. Melanogaster UAS-Mmp1^{RNAi}	Gift from D. Bohmann. (Mirka and Bohmann. 2006)	

Table S5 Primers Table

REAGENT or RESOURCE	SOURCE	IDENTIFIER
Oligonucleotides		
Primers for Rpl32.<br>F-AAGCGGCGACGCACTCTGTT<br>R-GCCCAGCATACAGGCCCAAG	This paper.	
Primers for Mmp1.<br>F-CTCCAACTGGGAGGGTGTTC<br>R-GAGTCAACAGCAAATCGGGC	This paper.	
Primers for Mmp2.<br>F-GGAGTCTCCAGTCTTTTGGCA<br>R-CCGCACCGAGGTTTCTGTAT	This paper.	
Primers for Delta.<br>F-TCGCTGGAGTACGATTTCCG<br>R-TGGCGCATTTGGGTATGTGA	This paper.	
Primers for Notch.<br>F-ACTAATACTGGCCGCTCGTCT<br>R-CGGTGTCTCATCCTTATCGTC	This paper	

Table S6 Antibodies Table

REAGENT or RESOURCE	SOURCE	IDENTIFIER
Antibodies		
Mouse monoclonal anti-LacZ (1/50)	DSHB	Cat#40-1a<br>RRID:AB_528100
Rabbit polyclonal anti-LacZ (1/2000)	MP Bio	Cat#559761<br>Lot#06825<br>RRID:AB_2687418
Mouse monoclonal anti-Repo (1/50)	DSHB	Cat#8D12<br>RRID:AB_528448
Alexa Fluor 647 Goat polyclonal anti-HRP (1/100)	Jackson ImmunoResearch	Cat#123-605-021<br>RRID:AB_2338967
Mouse monoclonal anti-Delta (1/100)	DSHB	Cat#C594.9B<br>RRID:AB_528194
Mouse monoclonal anti-MMP1 (1/100 of cocktail)	DSHB	Cat#3A6B4<br>RRID:AB_579780
Mouse monoclonal anti-MMP1 (1/100 of cocktail)	DSHB	Cat#3B8D12<br>RRID:AB_579781
Mouse monoclonal anti-MMP1 (1/100 of cocktail)	DSHB	Cat#5H7B11<br>RRID:AB_579779
Mouse monoclonal anti-DLG (1/500)	DSHB	Cat#4F3<br>RRID:AB_528203
Mouse monoclonal anti-Brp (1/50)	DSHB	Cat#nc82<br>RRID:AB_2314866
Mouse monoclonal anti-GluRIIA (1/250)	DSHB	Cat#8B4D2<br>RRID:AB_528269
Mouse monoclonal anti-tubulin	Sigma	Cat# T6074<br>RRID:AB_477582

Supplementary Movies

Movie S1-mCherry^{RNAi}. Time-lapse video of larval prep from *Moody-GAL4/UAS-mCherry*^{RNAi} subjected to high K+ HL3 saline. The images were captured at ~2.3 frames per second for 2 min.

Movie S2-Delta^{RNAi}. Time-lapse video of larval prep from *Moody-GAL4/UAS-Delta*^{RNAi} subjected to high K+ HL3 saline. The images were captured at ~2.3 frames per second for 2 min and exported.

Movie S3-Mmp1 het mCherry^{RNAi}. Time-lapse video of larval prep from *mmp1*$^{+/2}$*;Moody-GAL4/mCherry*^{RNAi} subjected to high K+ HL3 saline. The images were captured at ~2.3 frames per second for 2 min.

Movie S4-Mmp1 het Delta^{RNAi}. Time-lapse video of larval prep from *mmp1*$^{+/2}$*;Moody-GAL4/Delta*^{RNAi} subjected to high K+ HL3 saline. The images were captured at ~2.3 frames per second for 2 min.

Supplementary References

1. Tsurudome K, Tsang K, Liao EH, Ball R, Penney J, Yang JS, et al. The Drosophila miR-310 cluster negatively regulates synaptic strength at the neuromuscular junction. Neuron. 2010;68(5):879-93. doi: 10.1016/j.neuron.2010.11.016. PubMed PMID: 21145002; PubMed Central PMCID: PMCPMC3034365.

2. Tinevez JY, Perry N, Schindelin J, Hoopes GM, Reynolds GD, Laplantine E, et al. TrackMate: An open and extensible platform for single-particle tracking. Methods. 2017;115:80-90. doi: 10.1016/j.ymeth.2016.09.016. PubMed PMID: 27713081.
